# Hydration (H_2_O and D_2_O) Dictates the Stabilities and Conformational Entropy of Transthyretin Tetramers

**DOI:** 10.1021/jacs.5c11055

**Published:** 2025-09-18

**Authors:** Carter Lantz, Robert L. Rider, Syuan-Ting Kuo, Zhenyu Xi, Emily Burningham, Sangho D. Yun, Arthur Laganowsky, David H. Russell

**Affiliations:** Department of Chemistry, 14736 Texas A&M University, College Station, Texas 77843, United States

## Abstract

Transthyretin (TTR) is a 56 kDa tetrameric protein complex that plays a key role in transporting thyroxine and retinol. Despite its important biological role, TTR exhibits adverse effects, including disassembly into monomers, dimers, and trimers that then aggregate into disease-causing oligomers and fibrils. Hydration (referring to H_2_O or D_2_O) has been shown to have strong effects on TTR tetramer stabilities; however, probing how perturbation of hydration alters tetramer dynamics and stabilities is challenging. Here, we use variable-temperature (5–50 °C) electrospray ionization and ion mobility-mass spectrometry to better understand the influence of D_2_O and H_2_O hydration on the structures, stabilities, and dynamics of both wild-type TTR and several mutants (TTR^L55P^, TTR^V30M^, TTR^T119M^, and TTR^V122I^). Our findings include the following: (i) TTR tetramers in D_2_O have lower average charge states compared to those in H_2_O at temperatures between 5 and 50 °C; (ii) there are fewer disassembly products (monomers and dimers) in D_2_O compared to H_2_O at different temperatures; and (iii) the relative abundances of compact versus extended tetrameric forms are shifted in D_2_O compared to H_2_O. Results from hydrogen–deuterium exchange (HDX) combined with bottom-up proteomics of different TTR mutants are consistent with hydration in β-sheet and interface-forming regions providing tetramer stability. Additionally, HDX combined with bottom-up proteomics indicates that shifts in hydration at the interface are responsible for the observed differences between mutants. Overall, our results show that D_2_O significantly alters the TTR stabilities and conformational entropy, highlighting the important role that hydration plays in protein dynamics.

## Graphical abstract

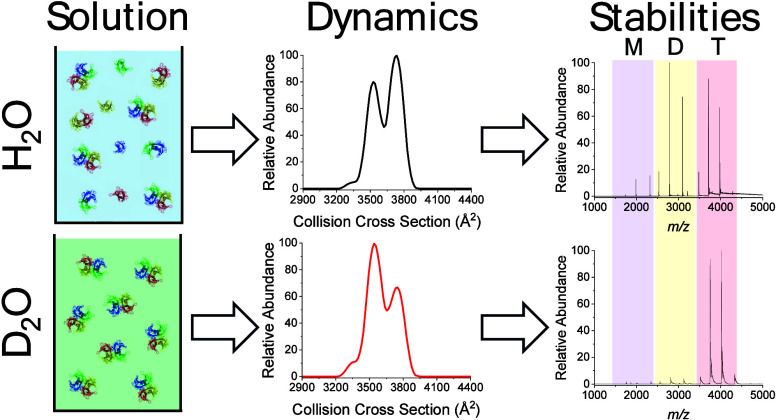

## Introduction

Transthyretin (TTR) tetramers have been extensively studied experimentally as well as with molecular dynamics simulations due to TTR’s unique dynamics and amyloidogenic nature.^1–4^ There are numerous point mutations that have been shown to alter tetramer stabilities and propensity to aggregate.^5^ The well-studied mutants TTR^L55P^, TTR^V30M^, and TTR^V122I^ exhibit an increase in aggregation rate relative to wild-type (wt) TTR.^6–8^ On the other hand, TTR^F87A^ and TTR^T119M^ show a decrease in aggregation rate because of their relative instability and stability, respectively.^9,10^ Environmental factors including ionic strength, pH, osmolytes, and metal ions shift dynamics and stabilities of TTR^9,11–13^ (i.e., the populations of tetramer conformations and microstates that make up the “rugged energy landscape” (REL)^14–16^ ). Some states are prone to disassembly and form monomers, dimers, and trimers that then reassemble to form higher-order oligomers.^17–20^ Mapping the distribution of conformations and microstates that populate the REL could lead to a greater understanding of the role of water (H_2_O or D_2_O) molecules in TTR complex stabilities and provide insight into TTR disassembly and aggregation.

Water molecules contribute to protein complex dynamics and stabilities;^21–29^ however, not all water molecules behave in a similar manner. Some are described as “cold water” while others are described as “hot water”, designations that infer dynamics rather than temperature. Other studies have referred to this phenomenon as “the two kinds of water,”^3^ “the two roles of water,”^21^ and “the two faces of water”.^30^ Cold water plays important roles in stabilizing protein structure and modulating ligand binding by interacting with polar amino acid side chains and the backbone and slowly exchanging with bulk water.^21,31–34^ At cold temperatures, water molecules hydrate hydrophobic regions of proteins and promote restructuring.^35–37^ Cold water molecules are observed in crystal structures of TTR; specifically, they are located in the dimer interface region of TTR tetramers.^38–40^ In fact, one study reported that eight cold water molecules are integral to TTR structure and stabilities.^41^ Disruption of cold water networks could alter the TTR structure, which would shift the REL and promote tetramer disassembly and formation of oligomers. For example, lowering the pH of TTR solutions promotes tetramer disassembly and favors oligomer and/or fibril formation.^17,18^ Other studies have reported that a decrease in solution temperature promotes disassembly of TTR tetramers, thereby promoting subunit exchange.^12,19,34,42^ While these studies underscore the important roles that hydration plays, the underlying mechanisms are unclear.

Here, we compare the effects of H_2_O and D_2_O on wild-type (wt) and mutant (i.e., L55P, V30M, T119M, and V122I) TTR tetramers, which were selected because of their varying degrees of dynamics and stabilities. Moreover, the physiochemical properties of D_2_O (e.g., strength of hydrogen bonding, temperature-dependent viscosity, and ionic strength) differ from those of H_2_O,^43,44^ and some studies have shown that protein complex dynamics are altered in D_2_O.^45,46^ Recent molecular dynamic simulations show that proteins are more rigid in D_2_O,^47^ and another study showed that proteins are more stable in D_2_O than in H_2_O.^48^ Still, another study showed that TTR subunit exchange is slower in D_2_O compared to H_2_O;^49^ however, this same study determined that aggregation of deuterated TTR is similar to aggregation of the aggregation-prone TTR^L55P^ mutant.^49^ Other studies have determined that deuteration of protein complex nonexchangeable hydrogens influences both dynamics and stabilities.^48,50^ In this work, we compare average charge state (*Z*_avg_) measurements from variable-temperature electrospray ionization (vT-ESI) experiments and collision cross section (CCS) measurements from ion mobility-mass spectrometry (IM-MS) experiments to monitor TTR structural dynamics and conformer population abundances in D_2_O and H_2_O. Results from these studies show that TTR tetramer *Z*_avg_ and conformer abundance values shift in D_2_O compared to H_2_O. Moreover, results obtained from hydrogen–deuterium exchange (HDX) combined with bottom-up proteomics^51^ provide information on regions of the tetramer that are stabilized because of the presence of cold water molecules. Collectively, changes in *Z*_avg_ and conformer abundances in D_2_O but not in H_2_O (or vice versa) and differences in HDX underscore the important role of hydration in TTR tetramer solvent accessible surface area (SASA), stabilities, and conformational entropy.

## Results

Previous studies have forwarded the idea that D_2_O shifts protein and protein complex dynamics,^45–50^ but the underlying reasons for these changes remain unclear. In this work, we performed a series of MS experiments to explore the shift in dynamics and stabilities of TTR in D_2_O compared to H_2_O. Native mass spectrometry (nMS), a technique that “freeze-dries” protein complexes in their solution state before MS analysis,^52^ was performed on TTR and mutant tetramers in H_2_O and D_2_O to confirm the presence of tetramers and the progression of the HDX reaction. We then evaluated TTR and mutant TTR tetramers in solutions of H_2_O and D_2_O using vT-ESI, a technique that alters the temperature of the protein solution directly in pulled capillaries.^53^ IM-MS was then used to determine whether differences in tetramer conformational entropy can be observed when TTR is electrosprayed in D_2_O. Lastly, HDX combined with bottom-up proteomics was utilized to locate regions of TTR tetramers that interact with cold water molecules that provide stability to tetramer complexes.

### nMS Analysis of Deuterated TTR

Figure 1 contains information about the mass of intact TTR tetramers after nMS was performed on tetramers incubated in H_2_O or D_2_O for 24 h. Mass spectra of solutions containing TTR show signals corresponding to the 13+, 14+, 15+, and 16+ charge states of tetrameric TTR (Figure 1A). Low abundance signals of other TTR species are observed in the spectra (e.g., monomers and octamers); however, charge states for these species indicate they were present in the solution and do not appear as a result of excess collisional activation. Tetramer signals are also well resolved, which indicates that most adducts were removed from TTR tetramers during ESI and that the HDX reaction is at a state of relative equilibrium. Tetramer signals were deconvoluted to obtain molecular weights for tetramers in H_2_O and D_2_O solutions (Figure S1). Tetramers in D_2_O increase in mass by ∼550 Da relative to H_2_O solutions (Figure 1B), which corresponds to ∼65% of the exchangeable hydrogen atoms on TTR (Figure 1C). nMS of TTR and mutant TTR solutions demonstrates that TTR tetramers can be electrosprayed in D_2_O and H_2_O without significant disassembly of TTR complexes and that a shift in mass can be observed.

**Figure 1:**
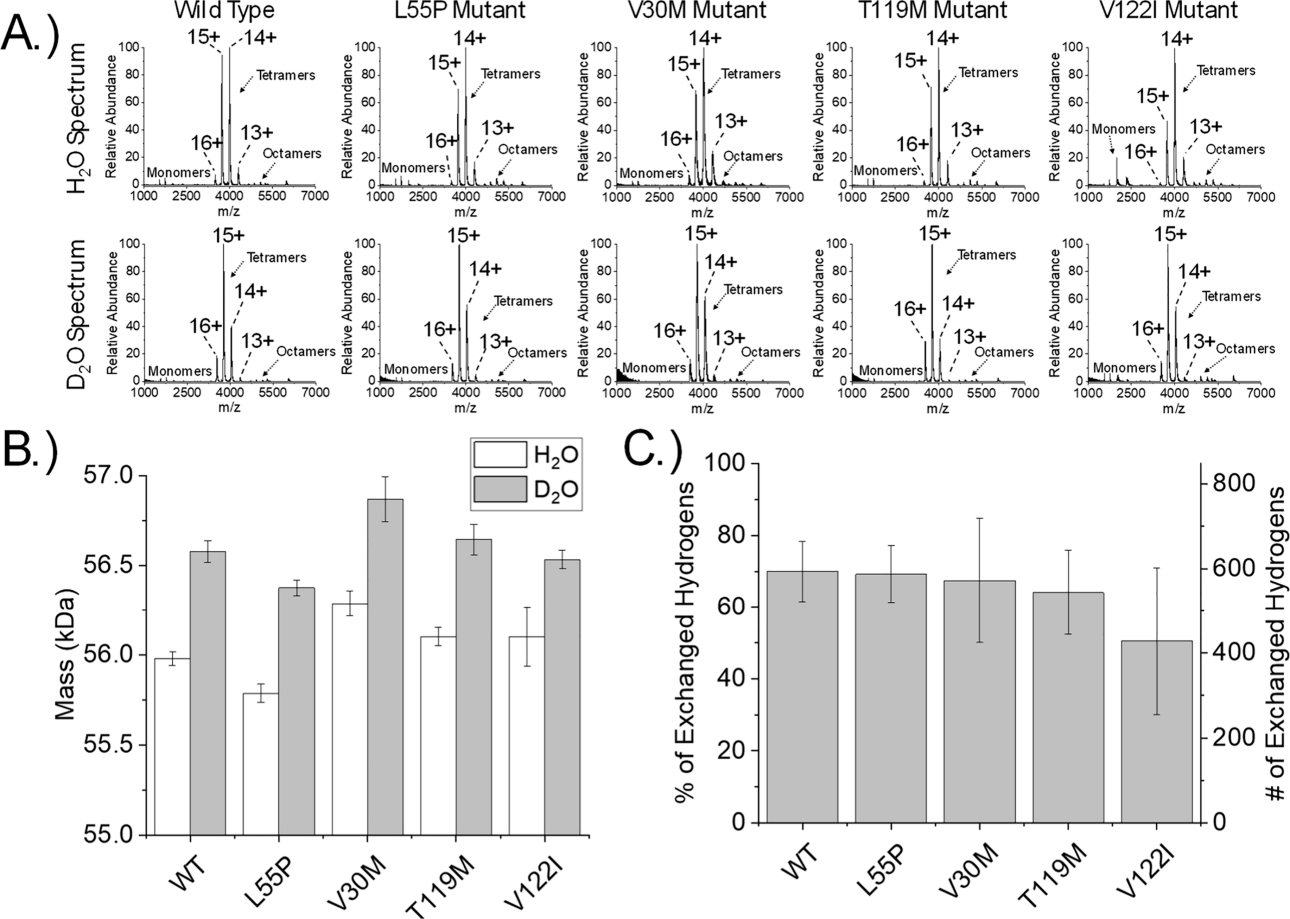
(A) nMS analysis of TTR, TTR^L55P^, TTR^V30M^, TTR^T119M^, and TTR^V122I^ tetramers electrosprayed in H_2_O or D_2_O with (B) the average molecular weight values and (C) the percentage of exchangeable hydrogen atoms exchanged for all tetramers. Reported are the mean and standard deviation (*n* = 5).

Figure 2 shows temperature-dependent *Z*_avg_ plots for TTR tetramers in H_2_O and D_2_O using vT-ESI. The plots show a *Z*_avg_ range of 14.2 to 15.1 with a minimum *Z*_avg_ value for all tetramers at 15 °C. We interpret shifts in *Z*_avg_ as evidence for hot and cold restructuring of TTR tetramers. This behavior is consistent with other research reported by Rossky and co-workers,^36^ Ben-Naim,^54^ and others^55–58^ that consider the effects of hot and cold solution environments on protein dynamics. It is generally agreed that shifts in *Z*_avg_ are evidence for conformation changes analogous to changes in SASA (isoelectric points),^53,59^ which is consistent with hydration altering TTR dynamics and stabilities.^36,60^ Differences in the tetramer *Z*_avg_ values can be observed in D_2_O relative to H_2_O in the vT-ESI experiments. For TTR, a decrease in *Z*_avg_ is observed in D_2_O at low (5–10 °C) and high (30–50 °C) temperatures relative to the *Z*_avg_ in H_2_O (Figure 2A). A similar trend was observed for TTR^L55P^ (Figure 2B). In contrast, TTR^V30M^ showed a decrease in *Z*_avg_ in D_2_O at low temperatures (5–10 °C) compared to *Z*_avg_ in H_2_O, but no shift at high temperatures (Figure 2C). Meanwhile, TTR^T119M^ (Figure 2D) and TTR^V122I^ (Figure 2E) exhibited no appreciable differences in *Z*_avg_ in D_2_O at any temperature relative to H_2_O. Observed differences in *Z*_avg_ are compelling evidence that D_2_O shifts TTR tetramer SASA relative to H_2_O and that the response to D_2_O varies among different TTR tetramers.

**Figure 2:**
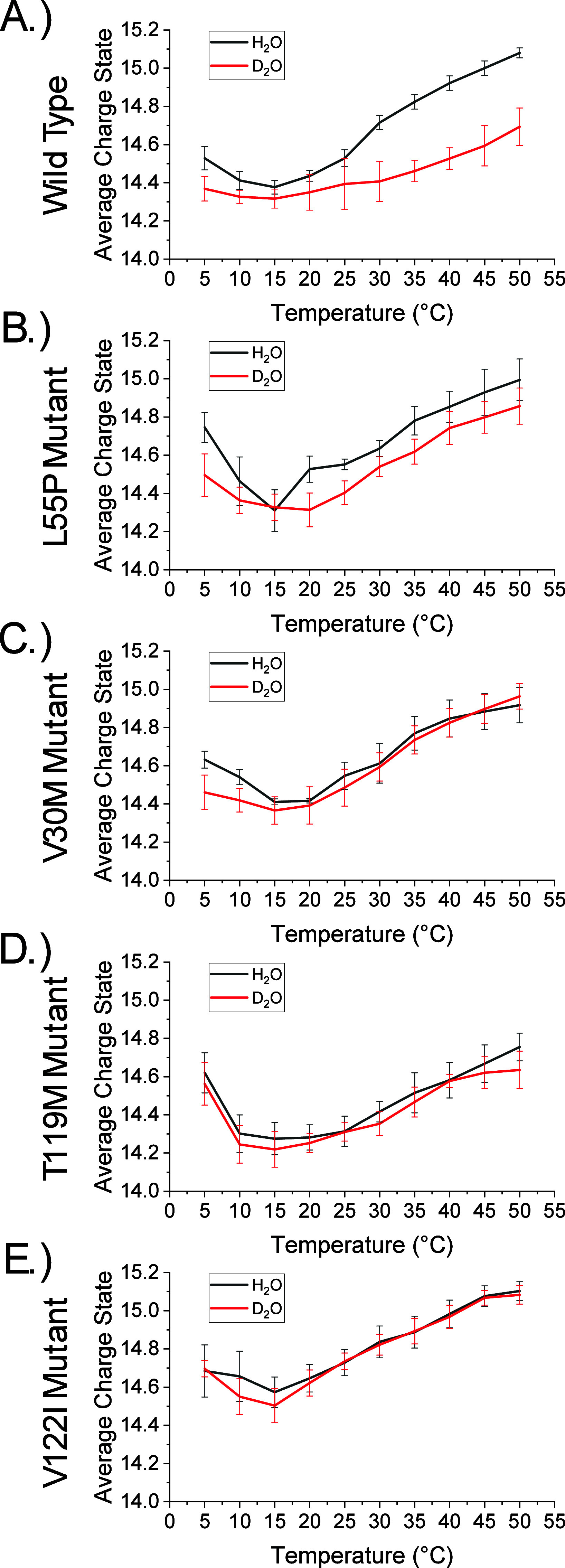
VT-ESI analysis for (A) TTR, (B) TTR^L55P^, (C) TTR^V30M^, (D) TTR^T119M^, and (E) TTR^V122I^ tetramers showing decreases in *Z*_avg_ in D_2_O relative to H_2_O, which indicate changes in SASA. Reported are the mean and standard deviation (*n* = 6–7).

It is important to note that at many temperature values, monomers and dimers are present in the mass spectra (Figures S2–S6). TTR and mutant TTR solutions in H_2_O yield abundant dimers and monomers at low and high temperatures. The notable exception is TTR^T119M^, which did not disassemble into abundant dimers in H_2_O at any temperature (Figure S5). Low dimer abundance for TTR^T119M^ is consistent with previous literature stating that TTR^T119M^ is more stable relative to the other mutants studied due to its ability to recruit water molecules to the tetramer interface.^3,10,34^ Dimers and monomers are present for TTR and mutant TTR tetramers in D_2_O, but abundances for those species diminish relative to their abundance in H_2_O. Decreases in the abundances of monomers and dimers in D_2_O compared to H_2_O are consistent with increased TTR tetramer stabilities in D_2_O.

### D_2_O Shifts Conformer Abundances of TTR and Mutant TTR

IM-MS was performed on TTR tetramers at ambient temperature (∼22 °C) to determine if D_2_O alters TTR conformational entropy, and the results for the 13+ and 14+ charge states are included in Figure 3. Data for the 15+ and 16+ charge states are also provided (Figure S7). Data was only collected at ambient temperature because we have previously reported that TTR tetramers do not thermally unfold in the temperature range of our experiments (Figure S8).^58^ CCS measurements for TTR in H_2_O reveal three conformers for the 13+ charge state and the 14+ charge state (Figure 3A and Figure S9 and Table S1). Abundances of these conformers in D_2_O shift to smaller CCS values, especially for the 14^+^ charge state. IM-MS data for TTR^L55P^ tetramers are similar to those for TTR (Figure 3B and Figure S10 and Table S2). Conformer abundances for the 13+ and 14+ charge states of TTR^L55P^ in H_2_O are similar to those for TTR in H_2_O. In D_2_O, smaller TTR^L55P^ conformers increase in abundance for the 13^+^ and 14^+^ charge states compared to H_2_O. IM-MS profiles for the 15+ and 16+ charge states of TTR (Figures S7A and S9 and Table S1) and TTR^L55P^ (Figures S7B and S10 and Table S2) do not shift significantly in D_2_O compared to H_2_O. The exception is 15+ of TTR^L55P^, which shows a small increase in abundance of the smaller conformer. We interpret shifts in conformer abundances in D_2_O as evidence for TTR tetramer restructuring in D_2_O, which is consistent with hydration having strong effects on both TTR and TTR^L55P^ conformational entropy.

**Figure 3:**
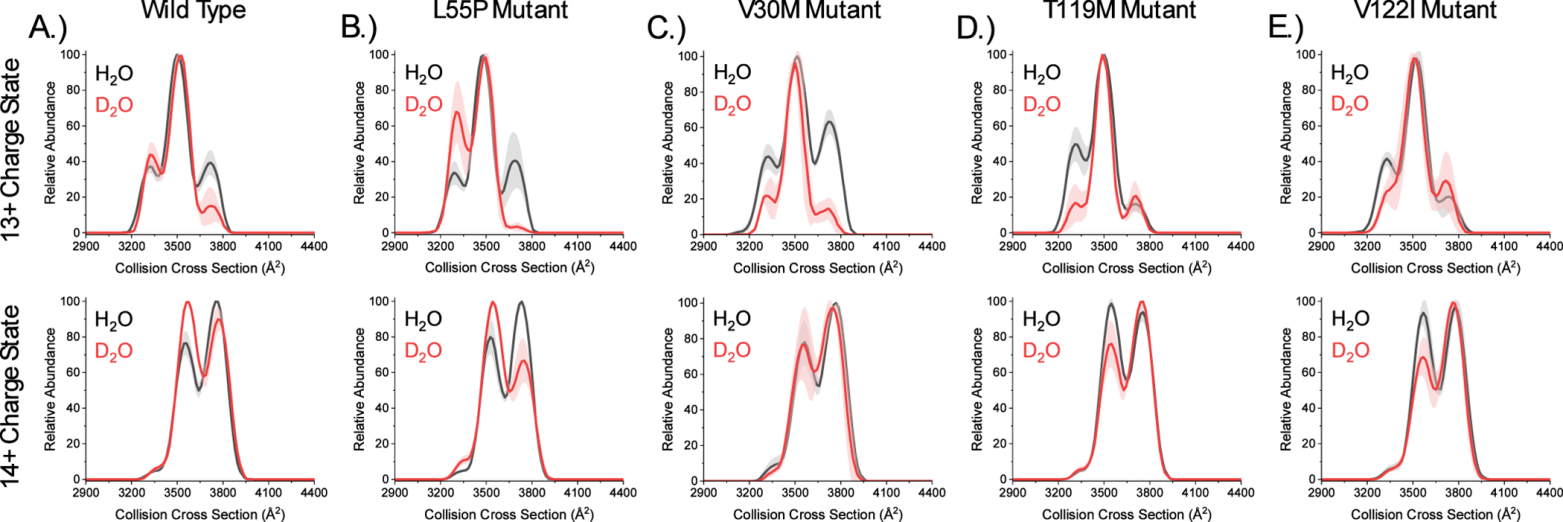
CCS plots for the 13+ and 14+ charge states of (A) TTR, (B) TTR^L55P^, (C) TTR^V30M^, (D) TTR^T119M^, and (E) TTR^V122I^ tetramers electrosprayed at ambient temperature in H_2_O and D_2_O. The mobilograms show that when electrosprayed in D_2_O, TTR conformers change in abundance. We interpret differences in conformer abundance as evidence that hydration with D_2_O alters TTR tetramer conformational entropy relative to H_2_O. Reported are the mean and standard deviation (*n* = 5).

Effects of D_2_O and H_2_O on TTR^V30M^, TTR^T119M^, and TTR^V122I^ are quite different from those observed for TTR and TTR^L55P^. Conformer abundances for TTR^V30M^ in H_2_O are most similar to conformer abundances for TTR and TTR^L55P^ in H_2_O (Figure 3C); however, in D_2_O, conformer abundances for the 13+ charge state of TTR^V30M^ shift to favor intermediate conformers, and conformer abundances for the 14+ charge state do not shift significantly (Figure 3C and Figure S11 and Table S3). For both TTR^T119M^ (Figure 3D and Figure S12 and Table S4) and TTR^V122I^ (Figure 3E and Figure S13 and Table S5), conformer abundances in H_2_O shift to favor smaller CCS conformers relative to TTR, TTR^L55P^, and TTR^V30M^, although, in D_2_O, larger CCS conformers for both the 13+ and 14+ charge states are favored for TTR^T119M^ and TTR^V122I^ relative to their abundance in H_2_O. Conformer abundances for the 15+ and 16+ charge states do not shift in D_2_O for TTR^V30M^ (Figures S7C and S11 and Table S3), TTR^T119M^ (Figures S7D and S12 and Table S4), or TTR^V122I^ (Figures S7E and Figure S13 and Table S5). Different conformer abundance shifts in D_2_O for TTR^V30M^, TTR^T119M^, and TTR^V122I^ relative to those for TTR and TTR^L55P^ indicate that not all tetramers respond in the same way to changes in hydration with D_2_O.

### How Might the Roles of Cold Water on TTR Stabilities and Dynamics Be Deciphered?

The data presented thus far show that shifts in hydration alter TTR tetramer dynamics, but the details regarding those changes in hydration are not clear. To glean information on specific regions containing water molecules that contribute to observed *Z*_avg_ and conformer abundance differences, conventional HDX combined with a bottom-up proteomics approach was performed. HDX combined with bottom-up proteomics reveals shifts in amounts of hydrogen atoms exchanged for deuterium atoms, which provides insight into alterations in protein dynamics and stabilities. We aimed to use HDX combined with bottom-up proteomics on TTR tetramers to infer which TTR regions interact with hot water molecules and which TTR regions interact with cold water molecules. Importantly, while HDX combined with bottom-up proteomics does not directly reflect the chemical exchange rates of hot versus cold water, it remains a valuable tool for probing protein dynamics and stabilities modulated by those water molecules whose presence is supported by crystallographic studies.^38,41,61^

HDX combined with bottom-up proteomics was performed on TTR, TTR^L55P^, TTR^V30M^, and TTR^V122I^ by incubating tetramers in D_2_O at ambient temperature (∼22 °C) for 24 h, and the results are included in Figure 4. TTR^T119M^ was excluded because it could not be digested under the low pH conditions, possibly due to reduced β-sheet structural fluctuations for the T119M mutant.^62,63^ Peptides 1–15, 21–29, 41–63, 72–79, 80–93, and 122–129 showed an average HDX percentage of over 91% (Figure 4A and Table 1), with some peptides reporting over 100% HDX due to back exchange that occurred during the peptide mapping experiment. The relatively high degree of HDX for these peptides is consistent with these regions having access to bulk hydration and readily exchanging hydrogen with deuterium. The TTR crystal structure reveals that these regions correspond to the solvent-exposed *N*-terminus and loop regions of the tetramer that do not interact with water molecules that provide tetramer stability (Figure 4B and Figure S14). In contrast, peptides 16–29, 30–40, 66–77, 94–110, and 111–121 had HDX values below 91% (Figure 4A; Table 1), which is consistent with these peptides having less access to deuterium from D_2_O. The TTR crystal structure reveals that these regions correspond to interior β-sheet regions and the C-terminus that forms the dimer/dimer interface (Figure 4B and Figure S14). We interpret the low HDX value for these regions as evidence that these regions contain cold water molecules that provide tetramer stability. Our findings, which pinpoint regions with higher dynamics and regions potentially stabilized by cold water molecules, align with a previous NMR study using HDX to probe TTR structure.^39^ Hydration shifts in regions containing cold water molecules could significantly impact TTR tetramer dynamics and contribute to differences in the stabilities and conformational entropy observed in this study.

**Table 1:** TTR Peptides Analyzed with HDX in this Study

amino acid number	TTR sequence
1 to 15	GSGPTGTGESKCPLM
16 to 29	VKVLDAVRGSPAIN
21 to 29	AVRGSPAIN
30 to 40	VAVHVFRKAAD
41 to 63	DTWEPFASGKTSESGELHGLTTE
66 to 77	FVEGIYKVEIDT
72 to 79	KVEIDTKS
80 to 93	YWKALGISPFHEHA
94 to 110	EVVFTANDSGPRRYTIA
111 to 121	ALLSPYSYSTT
122 to 129	AVVTNPKE

**Figure 4:**
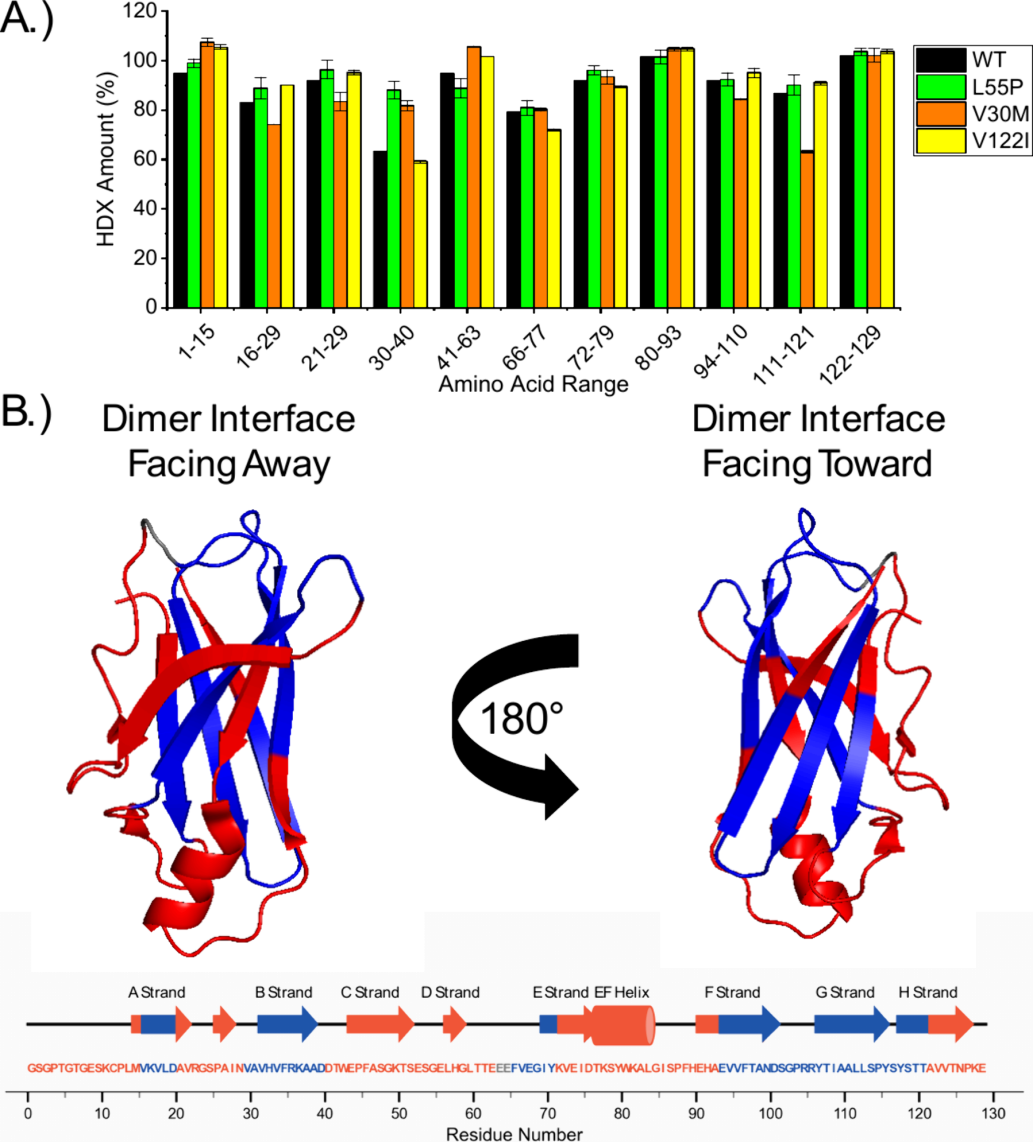
(A) The percentage of hydrogen atoms exchanged for deuterium atoms for various peptides of TTR, TTR^L55P^, TTR^V30M^, and TTR^V122I^ tetramers. Error bars reported are the mean and standard deviation (*n* = 2). Only one replicate could be collected for TTR. (B) Structure of the TTR monomer and a secondary structure scheme showing regions that exchange hydrogen atoms with deuterium atoms readily (red) and regions that do not exchange hydrogen atoms with deuterium atoms as readily (blue). The two glutamic acid residues (E64 and E65) that are not observed in any peptide are highlighted in gray. Adapted with permission from ref 61. Copyright 2000 Elsevier.

## Discussion

Results from this study illustrate that TTR tetramer dynamics and stabilities are in large part slaved to hydration.^64,65^ Specifically, vT-ESI and IM-MS reveal notable *Z*_avg_ and conformer abundance differences in D_2_O compared to H_2_O, which is consistent with hydration-dependent changes in stabilities and conformational entropy. HDX combined with bottom-up proteomics was utilized to determine that cold water molecules providing tetramer stability are located at the β-sheet and interface-forming regions of the tetramer. Here, we correlate shifts in hydration at the interface to differences in *Z*_avg_ and CCS profiles and hypothesize that differences in mutant dynamics are related to the location of the mutation in the tetrameric structure.

It is evident from vT-ESI and IM-MS experiments (Table 2) that hydration plays an important role in dictating the TTR and TTR mutant structural dynamics. Shifts in *Z*_avg_ are interpreted as changes in SASA, whereas shifts in CCS profiles report changes in the conformational entropy. TTR and the TTR^L55P^ mutant show decreases in *Z*_avg_ at low and high temperatures in D_2_O compared to H_2_O, indicating reduced SASA in D_2_O. These changes result from higher viscosity in D_2_O compared to H_2_O at low temperatures^64,66^ and shifts in salt interactions in D_2_O at higher temperatures.^67^ IM-MS data support the notion that TTR and TTR^L55P^ dynamics shift in D_2_O compared to H_2_O, as increased abundance of smaller CCS conformers is observed for both tetramers, which is consistent with compaction of their structure. Interestingly, compaction is greater for TTR^L55P^ compared to TTR, which correlates with TTR^L55P^ instability.^19,34^ In contrast, TTR^V30M^ shows a decrease in *Z*_avg_ only at low temperatures in D_2_O compared to H_2_O, which indicates that shifts in TTR^V30M^ SASA are more subtle. Additionally, IM-MS data for TTR^V30M^ show minimal changes in conformer abundance in D_2_O compared to H_2_O, which aligns with the minimal shift in *Z*_avg_ and correlates with its increased kinetic stability relative to TTR.^68,69^ TTR^T119M^ and TTR^V122I^ display another pattern, namely, *Z*_avg_ values do not shift significantly in D_2_O compared to H_2_O, and larger CCS conformers are observed in D_2_O. The trends for TTR^T119M^ and TTR^V122I^ indicate that their SASA does not change in D_2_O compared to H_2_O, yet their structure extends in D_2_O. Trends in *Z*_avg_ and CCS profiles across TTR tetramers show that hydration significantly influences tetramer SASA and conformational entropy, which affects their dynamics and stabilities.

**Table 2:** Summary of the *Z*_avg_ and CCS Data Shown in Figures 2 and 3

tetramer	decreased charge state in D_2_O at low temp (<20 °C)	decreased charge state in D_2_O at high temp (>20 °C)	compact structure in D_2_O (∼22 °C)	extended structure in D_2_O (∼22 °C)
wild type	Y	Y	Y	N
L55P mutant	Y	Y	Y	N
V30 M mutant	Y	N	N	N
T119 M mutant	N	N	N	Y
V122I mutant	N	N	N	Y

Mass spectra collected during vT-ESI experiments demonstrate that D_2_O increases TTR stabilities compared to H_2_O (Figures S2–S6). TTR, TTR^L55P^, TTR^V30M^, and TTR^V122I^ tetramers disassemble into abundant dimers and monomers in H_2_O at low temperatures. At high temperatures in H_2_O, dimers are less abundant but monomers are still present. The presence of abundant dimers and monomers in H_2_O at low temperatures is consistent with previous research showing that TTR disassembles readily at low temperatures.^9,18,34^ In D_2_O solutions, dimers and monomers are present, but they are less abundant compared to their abundance in H_2_O solutions at the same temperature. Previous studies have reported that proteins are more rigid in D_2_O relative to H_2_O,^47,70^ which may contribute to increases in stability of TTR tetramers in D_2_O solutions. Another study reported that subunit exchange of TTR subunits in D_2_O is slower because TTR disassembly is less apparent in D_2_O.^49^ We interpret decreases in monomer and dimer abundance in D_2_O compared to H_2_O in our data as evidence that TTR tetramer stabilities increase in D_2_O.

We link different mutant responses to D_2_O to shifts in dynamics of cold water at the tetramer interface,^46^ which are referred to as “bridging waters” because they span multiple subunits.^24,32^ Figure 5 displays differences in the percentage of hydrogen atoms exchanged for deuterium atoms for each mutant peptide relative to the corresponding TTR peptide. HDX at the C-terminus of TTR^L55P^ remains unchanged relative to that of TTR, showing similar interface stability and indicating that hydration at the interface is not disrupted. HDX at the C-terminus of TTR^V30M^ decreases, implying increased interface stability and stabilized hydration at the interface. Conversely, HDX at the C-terminus of TTR^V122I^ increases, which is consistent with a decreased interface stability and destabilized hydration at the tetramer interface. It is interesting to note that hydration at the N-terminus of the TTR mutants generally increases relative to TTR. Although this region does not interact directly with bridging water molecules, it is possible that changes in hydration at the N-terminus can shift the dynamics of the hydration shell, which could alter TTR dynamics and stabilities.^71,72^ Bridging water has been found to provide stability to TTR tetramers; therefore, we attribute changes in conformational entropy and stabilities for the different mutants to shifts in hydration at the tetramer interface.

**Figure 5:**
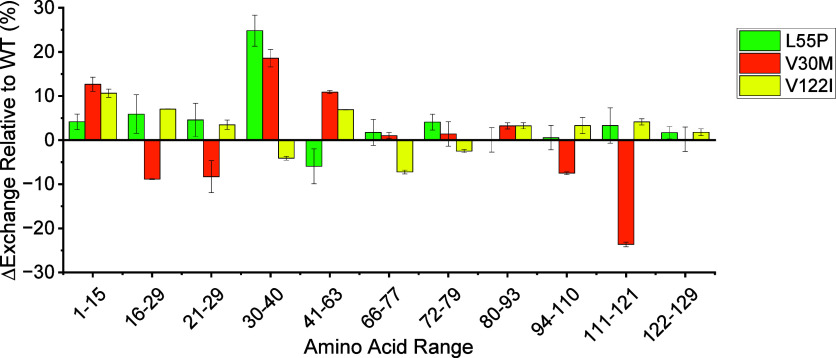
A plot showing the difference in HDX percentage for TTR^L55P^, TTR^V30M^, and TTR^V122I^ tetramers relative to that of TTR. Notice how different regions of the tetramer are perturbed by each mutation. Reported are the mean and standard deviation (*n* = 2).

TTR mutants alter hydration at the interface differently because of their location within the 3D TTR structure. As shown in Figure 6, the L55P mutation is situated in a solvent-exposed region and thus only interacts with bulk hydration, resulting in no significant change in hydration that stabilizes the tetramer interface, as illustrated by Figure 5. Because L55P does not perturb hydration at the interface, TTR^L55P^ has the same response to D_2_O relative to that of TTR (Table 2). In contrast, the V30M mutation resides in the β-sheet region of the TTR tetramer, which contains cold hydration. Alteration of cold hydration by V30M stabilizes bridging water molecules at the tetramer interface, which increases tetramer stability (Figure 5) and accounts for the reduced response of TTR^V30M^ to D_2_O relative to TTR (Table 2). The V122I mutation and T119M mutation are located directly at the interface of the tetramer and may increase hydration at the interface, as is evident for TTR^V122I^ in Figure 5. Increased hydration at the interface does not shift the SASA, but it extends the structure of these mutants (Table 2). Shifts in hydration at the interface caused by the mutations correlate with differences in *Z*_avg_ and CCS profiles seen in this study and may contribute to differences in the dynamics and stabilities that lead to tetramer disassembly and aggregation.

**Figure 6:**
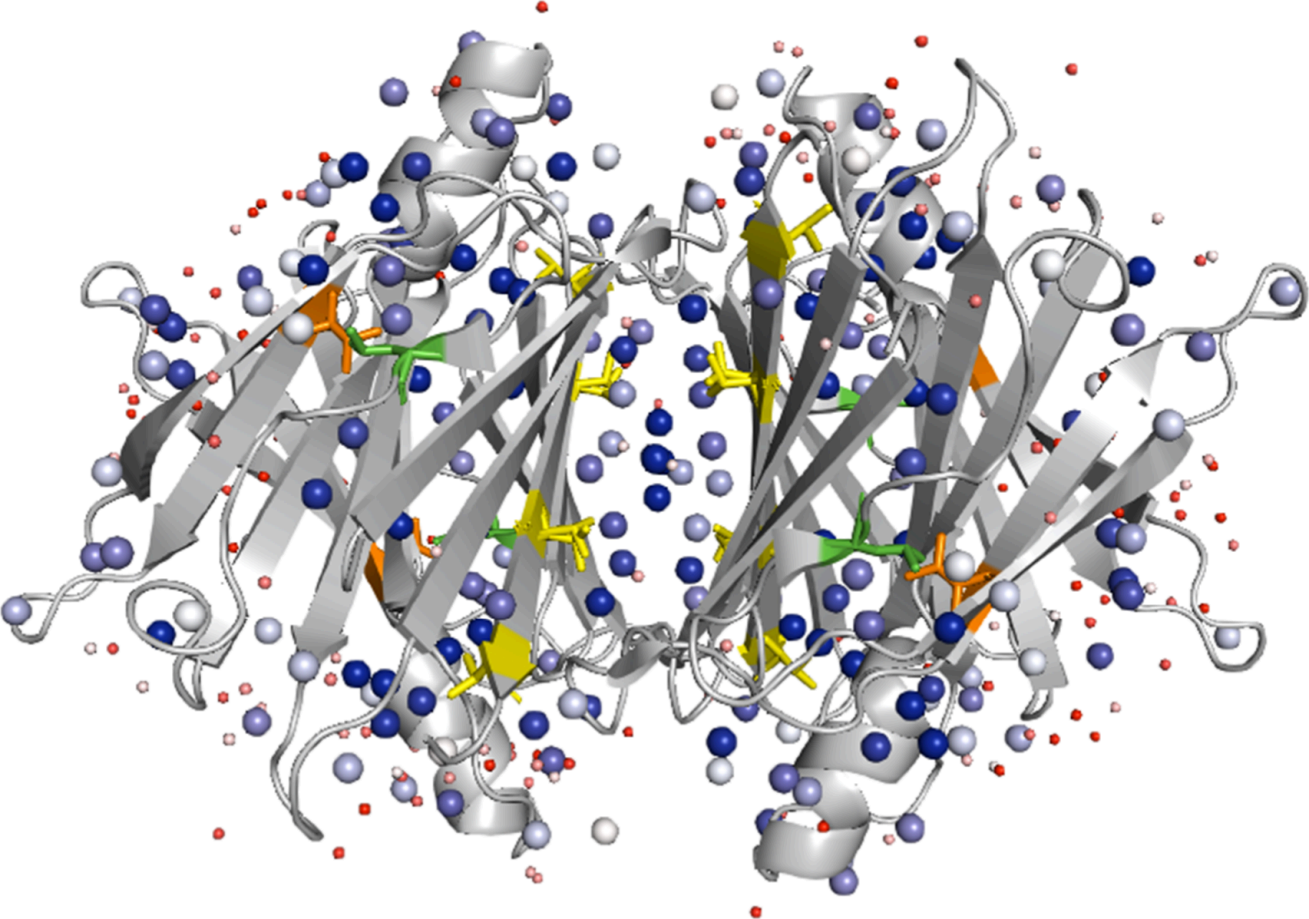
The crystal structure of TTR with mutants and H_2_O molecules is highlighted. TTR^L55P^ (green) is the mutant that shows decreases in *Z*_avg_ and compacts TTR structure in D_2_O; TTR^V30M^ (orange) is the mutant that shifts TTR *Z*_avg_ and structure only slightly in D_2_O; TTR^T119M^ and TTR^V122I^ (yellow) are the mutants that do not shift TTR *Z*_avg_ but do extend the TTR structure in D_2_O. Notice that the Z_avg_ and CCS data can be correlated with the location of the mutation on the structure. Spheres correspond to H_2_O molecules present in the crystal structure, and the color of the dots corresponds to the B-factor of the molecules with red being the most dynamic and blue being the least dynamic. More dynamic H_2_O molecules are reduced in size to emphasize the location of the less dynamic H_2_O molecules. Adapted with permission from ref 61. Copyright 2000 Elsevier.

It is worth noting that studies investigating how deuteration shifts protein dynamics report different conclusions, with some attributing observed effects to isotope effects, while others emphasize the role of hydration. Multiple studies have found that deuteration of nonexchangeable hydrogen atoms alters protein complex stabilities,^48,50^ and another study reported that deuterated TTR aggregates more rapidly than nondeuterated TTR.^49^ These data attribute altered protein stabilities to isotope effects rather than hydration effects. On the other hand, D_2_O and H_2_O have different bulk properties (Table 3),^46,73^ and properties that alter bulk hydration could shift protein dynamics and stabilities. Yee et al. showed that TTR subunit exchange is slower in D_2_O relative to H_2_O, which they attributed to hydration effects.^49^ Similarly, Haidar and Konermann reported that thermal unfolding in D_2_O is different for cytochrome *c*, lysozyme, and ubiquitin compared to H_2_O, but their collision-induced unfolding (CIU) profiles *in vacuo* did not shift, which is consistent with shifts in hydration rather than isotope effects altering thermal stabilities.^74^ We conducted a similar CIU experiment for the 14+ and 15+ charge states of TTR in H_2_O and D_2_O (Figure S15). The resulting CIU profiles and CIU_50_ values for gas phase ions did not shift in D_2_O compared to H_2_O, which demonstrates that isotope effects do not significantly affect TTR tetramer dynamics and stabilities. Thus, we conclude that TTR and mutant TTR SASA, stabilities, and conformational entropies are directly linked to hydration.

**Table 3:** Bulk Properties of H_2_O and D_2_O that Could Alter Hydration of TTR Tetramers (25 °C)

property	H_2_O	D_2_O
pH/pD	7.0	7.4
dynamic viscosity	1.00 mPa s	1.25 mPa s
dielectric constant	78.37	78.06
dielectric constant	3.59	3.76

## Conclusions

Here, we show *Z*_avg_, CCS, and data from HDX combined with bottom-up proteomics that indicate TTR tetramer SASA, stabilities, and conformational entropy shift in the presence of D_2_O compared to H_2_O. VT-ESI of TTR in D_2_O showed that decreases in *Z*_avg_ are apparent for TTR, TTR^L55P^, and TTR^V30M^ but are not apparent for TTR^T119M^ and TTR^V122I^. We interpret decreases in *Z*_avg_ as evidence that the TTR tetramer SASA decreases in D_2_O. VT-ESI of TTR tetramers at various temperatures is consistent with TTR being less prone to disassembly in D_2_O, as fewer dimers and monomers are present in D_2_O solutions. Thus, as noted by Sun et al., TTR tetramers are more stable in D_2_O.^49^ IM-MS analysis of TTR tetramers in H_2_O or D_2_O revealed that D_2_O compacts TTR and TTR^L55P^ structures, does not significantly alter TTR^V30M^ structure, and extends TTR^T119M^ and TTR^V122I^ structures. Additionally, HDX combined with bottom-up proteomics data revealed that hydration in β-sheet regions and the interface-forming region provide tetramer stability and shifts in that hydration at the interface-forming region could alter dynamics and stabilities of TTR tetramers.

Previous studies have forwarded the idea that “cold water molecules” (i.e., water molecules that interact with protein complexes and do not readily exchange with bulk water) integrate into TTR tetramer structure and enhance complex stability.^34,41^ Perturbation of cold water molecule structure alters the REL^14–16^ of TTR to favor the formation of non-native states that promote subunit exchange and aggregation. We interpret differences in TTR tetramer SASA, stabilities and conformational entropy in this study as evidence that the hydration of TTR tetramers with D_2_O alters their REL. HDX combined with bottom-up proteomics of different TTR mutants showed that shifts in dynamics and stabilities occur when mutations perturb cold hydration at the protein complex interface. It is possible that isotope effects could contribute to differences in stabilities for TTR tetramers; however, *in vacuo* data from Haidar and Konermann indicate that changes in protein stability in D_2_O are most likely related to hydration and not related to isotope effects.^74^ This study demonstrates that hydration shifts the REL of protein complexes and strongly indicates that it must not be ignored when making inferences about protein complex dynamics and stabilities.

## Experimental Section

### Sample Preparation

TTR and mutant TTR samples were expressed as described previously,^34^ and the samples were verified by high-resolution nano electrospray ionization (nESI) MS analysis. Two additional amino acids (Gly-Ser) remain on the *N*-terminus after removal of the *N*-terminal affinity tag with tobacco etch virus (TEV) protease. Ammonium acetate was added to solutions of H_2_O and D_2_O until the solution conductivity was 1.6–1.7 μS/cm (∼20 mM). An ammonium acetate concentration of 20 mM was utilized because TTR tetramers are more dynamic at low salt concentrations most likely due to fewer salt molecules interacting with TTR.^75,76^ Solutions of TTR, TTR^L55P^, TTR^V30M^, TTR^T119M^, and TTR^V122I^ were buffer exchanged into these solutions with Bio-Spin 6 SEC columns (BioRad, Hercules, CA) and incubated at ambient temperature for 24 h. Proteins were incubated for 24 h because it provided enough time for the HDX reaction to reach a relative state of equilibrium but did not allow enough time for exchange of protein hydrogen atoms that may shift the stabilities of TTR. Solutions were then diluted to 2.5–5 μM before analysis.

### Variable-Temperature Electrospray Ionization Mass Spectrometry Analysis

Solutions of TTR dissolved in D_2_O or H_2_O were inserted into gold-coated pulled electrospray capillaries, and a vT-ESI device described previously^53^ was utilized to vary the temperature of the solution in the nanospray tip. The solution was allowed 3 min to equilibrate at each temperature then electrosprayed with nESI on a Thermo Fisher Exactive Plus EMR Orbitrap mass spectrometer (Bremen, Germany). The source DC offset was set at 16 V, the injection flatapole DC was set at 10 V, the inter flatapole lens was set at 8 V, the bent flatapole DC was set from 5.25 to 6 V, the transfer multipole DC was set at 2 V, the C-trap entrance lens was set at 0 V, and the trapping gas parameter was set from 2 to 4. For sufficient desolvation of the tetramers, the in-source CE parameter was set to 25 V and the HCD energy given was 25 V. Per temperature point, 85–225 scans were collected. *Z*_avg_ values for each spectrum were calculated using UniDec.^77^ The mass range included only tetramer signals, and the mass was sampled every 1 Da. Error bars for vT-ESI experiments correspond to the standard deviation of 2 solutions prepped the same way using 3–4 nanospray tips each (i.e., 6–7 replicates).

### Ion Mobility-Mass Spectrometry Analysis

Solutions of TTR dissolved in D_2_O or H_2_O were inserted into the gold-coated pulled electrospray capillaries, and nESI was performed on the solutions with an Agilent 6560 ion mobility Q-ToF (Santa Clara, CA). Mass spectra were collected using 3-bit multiplexing mode for Hadamard processing.^78^ The electrospray voltage value was set at 1600 V, the in-source collision energy voltage was set to 55 V, and the drying gas value was set to 1.5. Nitrogen was utilized as the buffer gas. Data collection lasted 1.5–3 min. Data was demultiplexed with PNNL PreProcessor 4.1.^79^ All frames were compressed into one frame, and each drift bin was interpolated into three drift bins. Minimum pulse coverage was 100, and moving average smoothing was applied with a drift size of 3. Spike removal was also applied with 1 adjacent point per dimension, and the signal intensity lower threshold was set to 40 counts. Ion drift times were converted to CCS by using single field calibration. *Z*_avg_ values were calculated using UniDec.^77^ The *m*/*z* range was limited to include tetramer charge states; the mass was sampled every 1 Da; the signal fwhm parameter was set to 0.1 Th; and the beta value was set to 100. Mobilograms for each charge state were extracted from demultiplexed data. Error bars correspond to the standard deviation of 1 solution using 5 different nanospray tips. Conformer abundances were deconvoluted using OriginPro 2023 (Northampton, MA). The PDB code for structure analysis was 1F41.^61^ Collision-induced unfolding (CIU) experiments were performed using in-source collision energy ramped in 25 V increments from 0 to 400 V. Each sample was collected with three different nESI emitters. Drift time data was exported for the 14+ and 15+ charge state using the Agilent IM-MS Browser for each collision energy. CIU heatmaps were made using CIUSuite3 software^80^ with 5 smoothing window size 2D Savitzky-Golay smoothing. CIU_50_ values were extracted by using the standard fitting routine. Standard feature detection parameters were 2 minimum feature length, 0 maximum CV gap length, and 100 feature allowed width. Standard CIU_50_ parameters used were max spectral centroiding mode, 0 maximum CV gap length, and 200 transition region padding.

### Hydrogen–Deuterium Exchange Data Collection

For bottom-up deuterium uptake experiments, 6 μM transthyretin variants were labeled in 90% D_2_O with 20 mM MOPS and 180 mM NaCl (pH_read_ 6.8) for 24 h at 25 °C. Fully deuterated controls (All-D) were prepared by incubating samples in 7.2 M deuterated urea with 90% D_2_O (pH_read_ 6.8) for 24 h at 25 °C. Labeling was quenched with precalibrated 4 M guanidinium chloride and 0.1% TFA to achieve pH_read_ 2.45. Deuterium uptake percentage was normalized according to the following formula:

$$\mathrm{Deuterium}\;\mathrm{Uptake}\;(24\;\mathrm{hours})(\%)=\frac{D_{24\;\mathrm{hours}}-D_{\mathrm{All}-H}}{D_{\mathrm{All}-D}-D_{\mathrm{All}-H}}$$ 1

Online digestion and peptide separation were performed at 0–4 °C using a home-built chill box.^81^ Quenched samples were injected onto an immobilized acid protease column for online digestion, followed by peptide desalting and enrichment on a C_8_ trap column (5 μm 5 × 1.0 mm, Higgins Analytical) using a Vanquish Binary Pump from Thermo Fisher (San Jose, CA) at flow rate of 0.125 mL mL^–1^ with 0.4% formic acid (pH 2.6). Peptide separation was achieved using a C18 analytical column (3 μm 50 × 1.0 mm, Higgins Analytical) on a Vanquish Neo UHPLC system from Thermo Fisher (San Jose, CA) at flow rate of 50 μL mL^–1^ with mobile phases A (0.4% formic acid in water) and B (0.4% formic acid in 80% acetonitrile). The gradient was 2 to 60% B over 30 min, followed by a 30 min wash.

MS analysis was conducted using a Thermo Fisher Orbitrap Fusion Tribrid Mass Spectrometer (San Jose, CA) with the following ion source settings: spray voltage 3500 V, sheath gas 25 (arb. unit), auxiliary gas 5 (arb. unit), sweep gas 0 (arb. unit), ion transfer tube temperature 275 °C, and vaporizer temperature 75 °C. Hydrogen exchange MS data were acquired in full-scan (MS1) mode at a resolution of 120,000 (positive ion mode, RF lens 60%, and scan range *m*/*z* 350–2000). Peptide mapping was performed using data-dependent acquisition (DDA) with quadrupole isolation (1.2 *m*/*z* window), HCD activation (normalized collision energy 30%), 1 microscan, and orbitrap resolution of 30,000. Peptide identification was done using Proteome Discoverer 2.4 from Thermo Fisher (San Jose, CA) with MS1 and MS2 tolerances of 10 ppm and 0.4 Da, respectively. Deuterated peptide masses were extracted using EXMS 2 ^82^ with an MS1 tolerance of 10 ppm.

## Supplementary Material



## Notes

Additional data for variable-temperature electrospray ionization experiments and ion mobility-mass spectrometry experiments (PDF)
