# Supplementary material for: Hydration (H2O and D2O) Dictates the Stabilities and Conformational Entropy of Transthyretin Tetramers
Source: J Am Chem Soc. 2025 Sep 18;147(39):35616–26. doi: 10.1021/jacs.5c11055 (PMC12498396; doi:10.1021/jacs.5c11055)
Supplement: Supplementary file 1 [file ja5c11055_si_001.pdf]

Supporting Information

Hydration (H_2O and D_2O) Dictates the Stabilities and Conformational Entropy of Transthyretin Tetramers

Carter Lantz, Robert L. Rider, Syuan-Ting Kuo, Zhenyu Xi, Emily Burningham, Sangho D. Yun, Arthur Laganowsky, David H. Russell*

Department of Chemistry, Texas A&M University, College Station, TX 77843

*Corresponding author; Email: russell@chem.tamu.edu

Table of Contents

	Page
Figure S1	S2
Figure S2	S2
Figure S3	S3
Figure S4	S4
Figure S5	S5
Figure S6	S6
Figure S7	S7
Figure S8	S7
Figure S9	S8
Figure S10	S9
Figure S11	S10
Figure S12	S11
Figure S13	S12
Figure S14	S13
Figure S15	S13
Table S1	S14
Table S2	S14
Table S3	S15
Table S4	S15
Table S5	S16

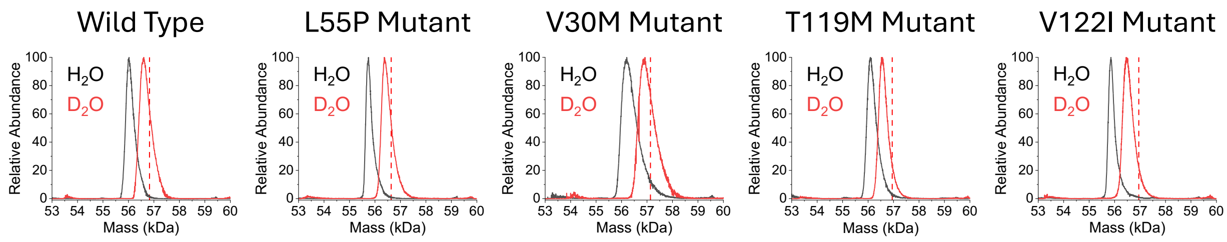

Figure S1: Deconvoluted spectra of the mutants analyzed in this study. The dotted red line represents the mass at which 100% of the exchangeable hydrogens have been replaced with deuterium on the protein.

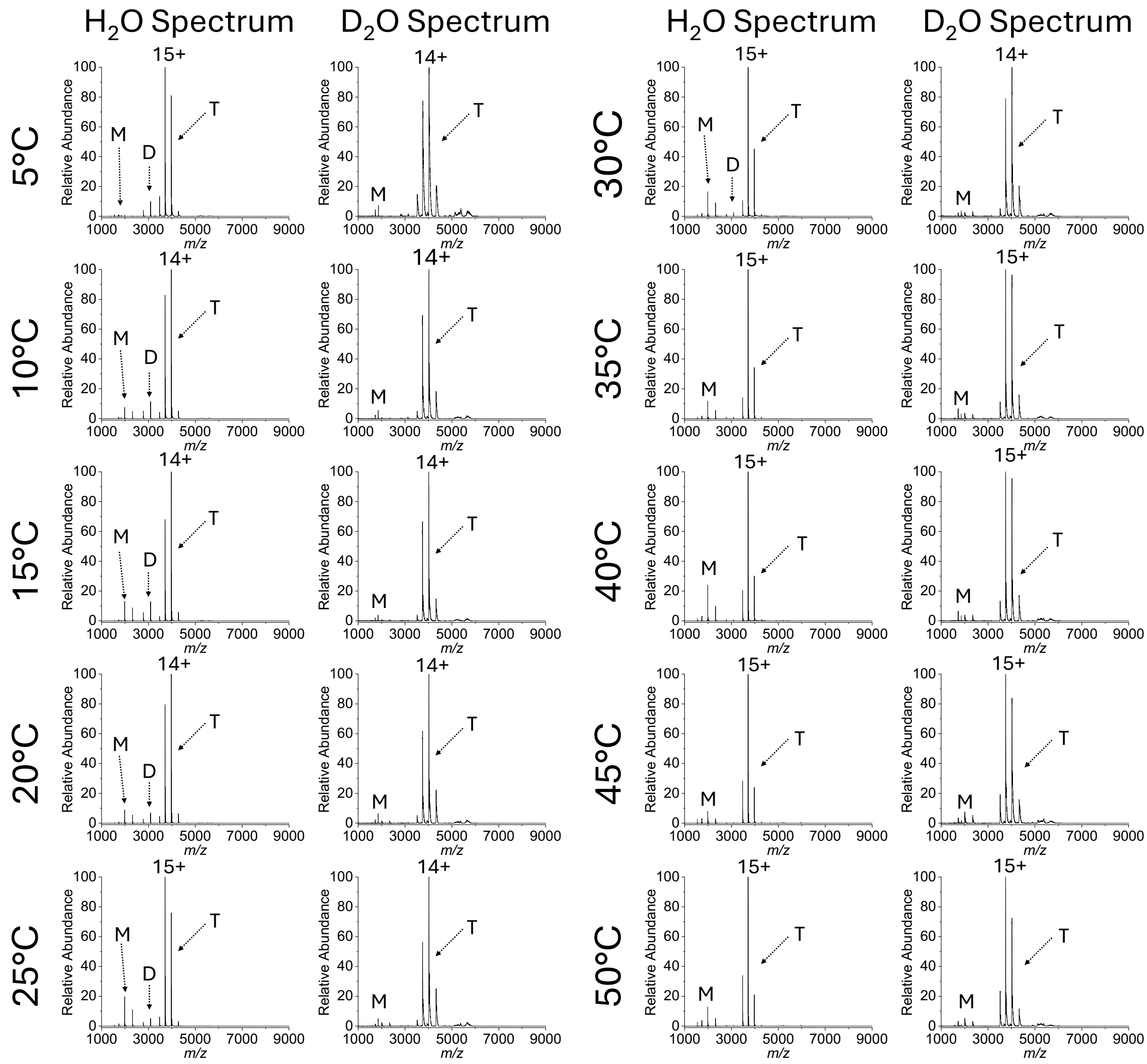

Figure S2: vT-ESI spectra (5-50 °C) for TTR in H_2O and D_2O with 20 mM ammonium acetate. (T) corresponds to tetramers, (D) corresponds to dimers, and (M) corresponds to monomers.

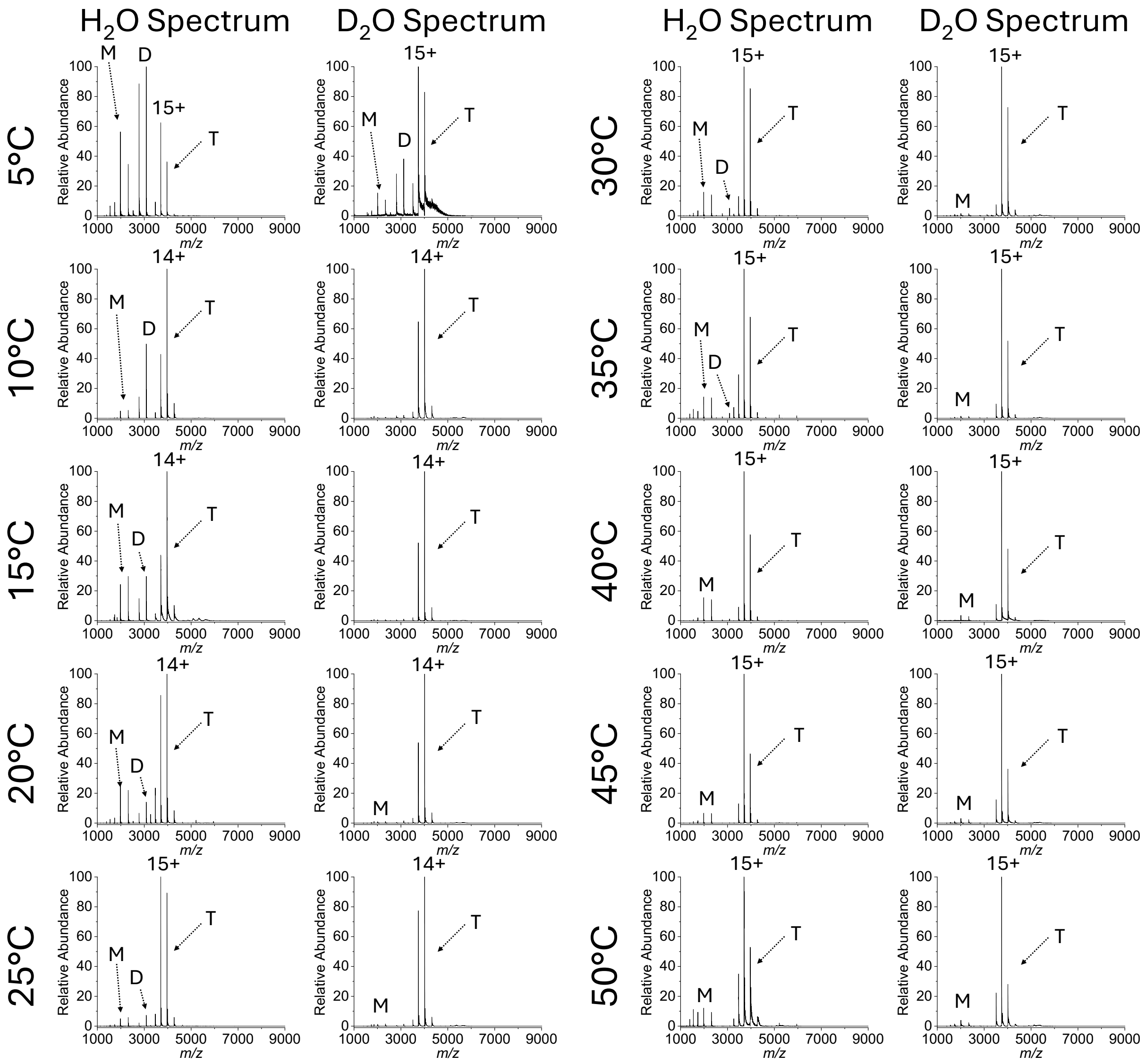

Figure S3: vT-ESI spectra (5-50 °C) for TTR^{L55P} in H_2O and D_2O with 20 mM ammonium acetate. (T) corresponds to tetramers, (D) corresponds to dimers, and (M) corresponds to monomers.

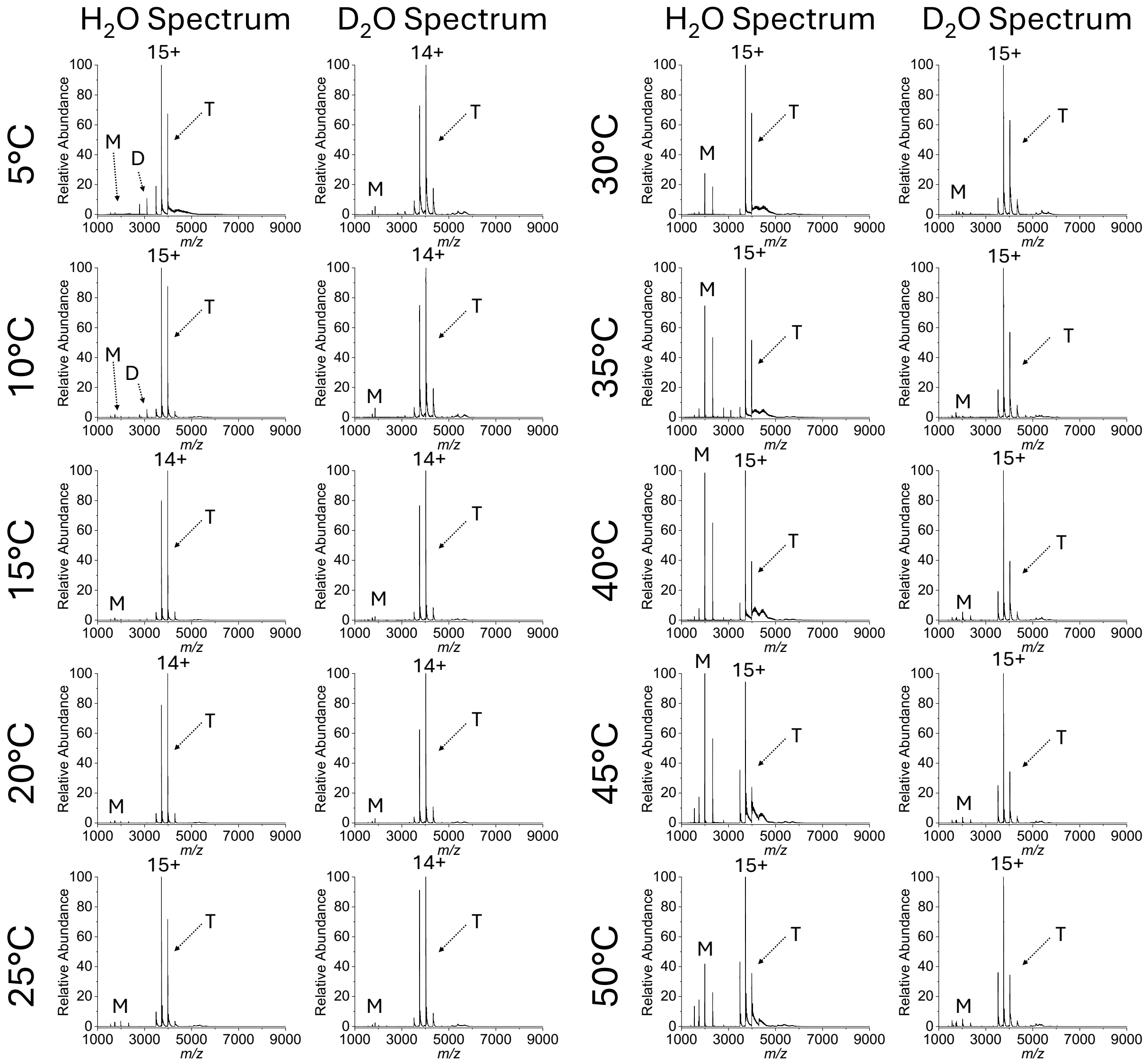

Figure S4: vT-ESI spectra (5-50 °C) for TTR^{V30M} in H_2O and D_2O with 20 mM ammonium acetate. (T) corresponds to tetramers, (D) corresponds to dimers, and (M) corresponds to monomers.

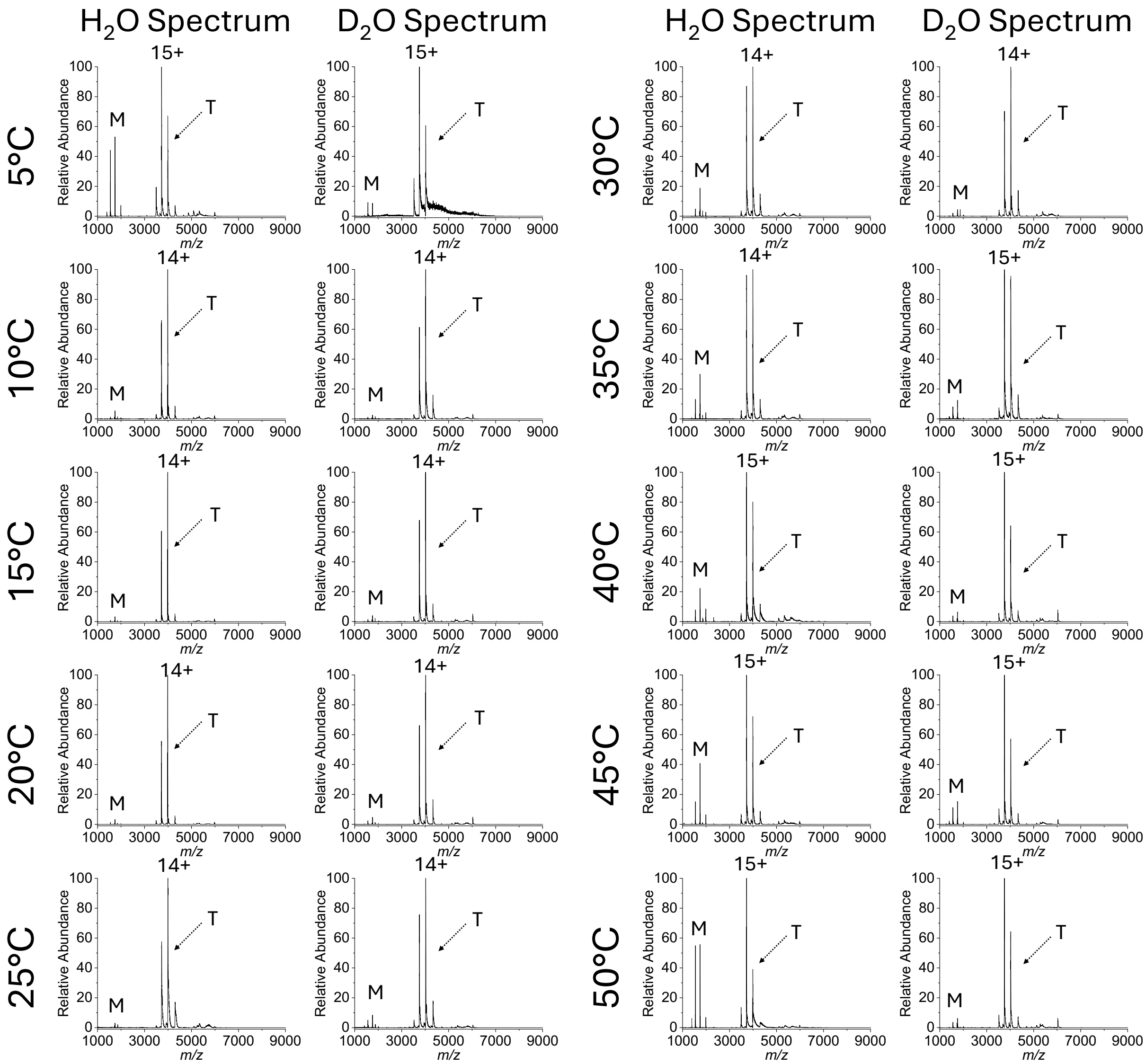

Figure S5: vT-ESI spectra (5-50 °C) for TTR^{T119M} in H_2O and D_2O with 20 mM ammonium acetate. (T) corresponds to tetramers, (D) corresponds to dimers, and (M) corresponds to monomers.

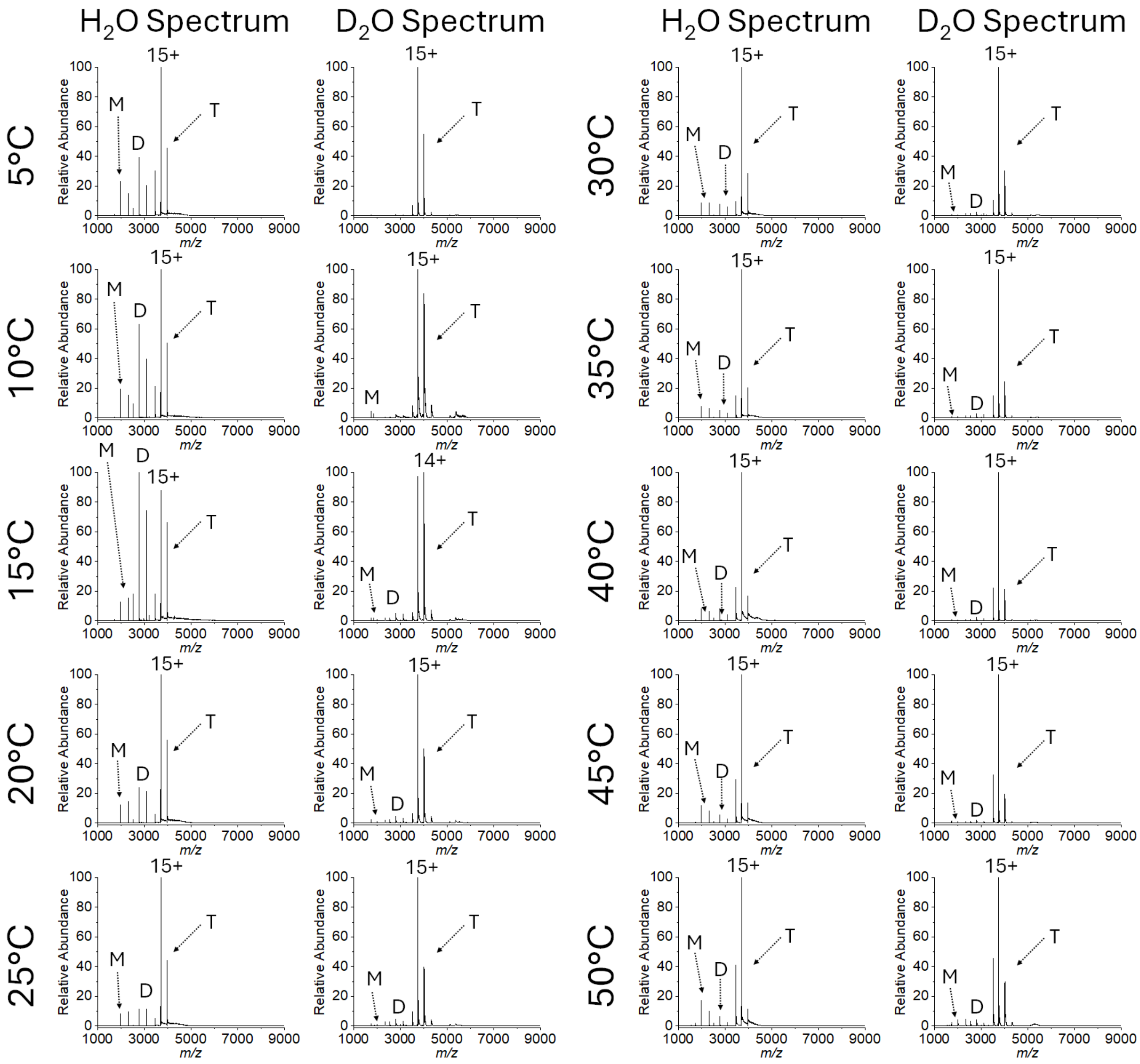

Figure S6: vT-ESI spectra (5-50 °C) for TTR^{V122I} in H_2O and D_2O with 20 mM ammonium acetate. (T) corresponds to tetramers, (D) corresponds to dimers, and (M) corresponds to monomers.

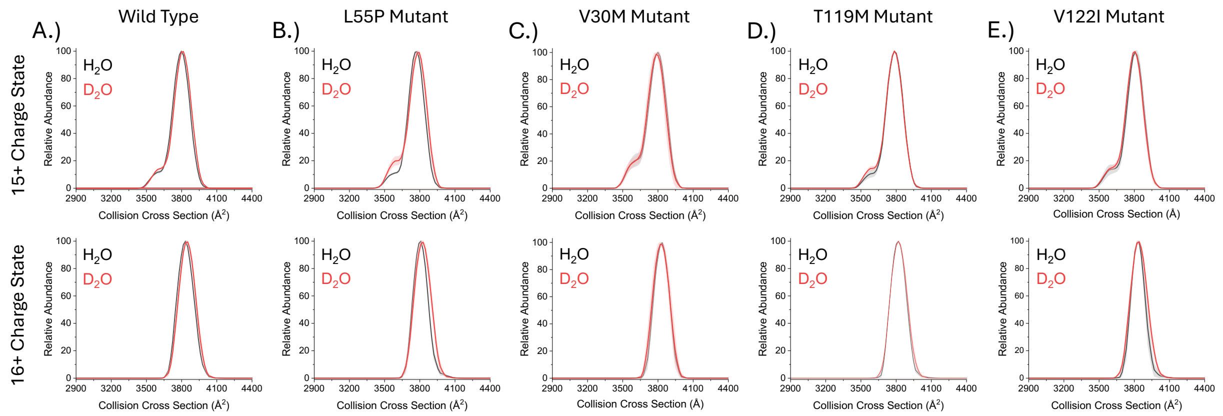

Figure S7: Mobilograms of the 15⁺ and 16⁺ charge state for A.) TTR, B.) TTR^{L55P}, C.) TTR^{V30M}, D.) TTR^{T119M}, E.) TTR^{V122I} tetramers when they are dissolved in H_2O and D_2O. Reported are the mean standard deviation (n=5).

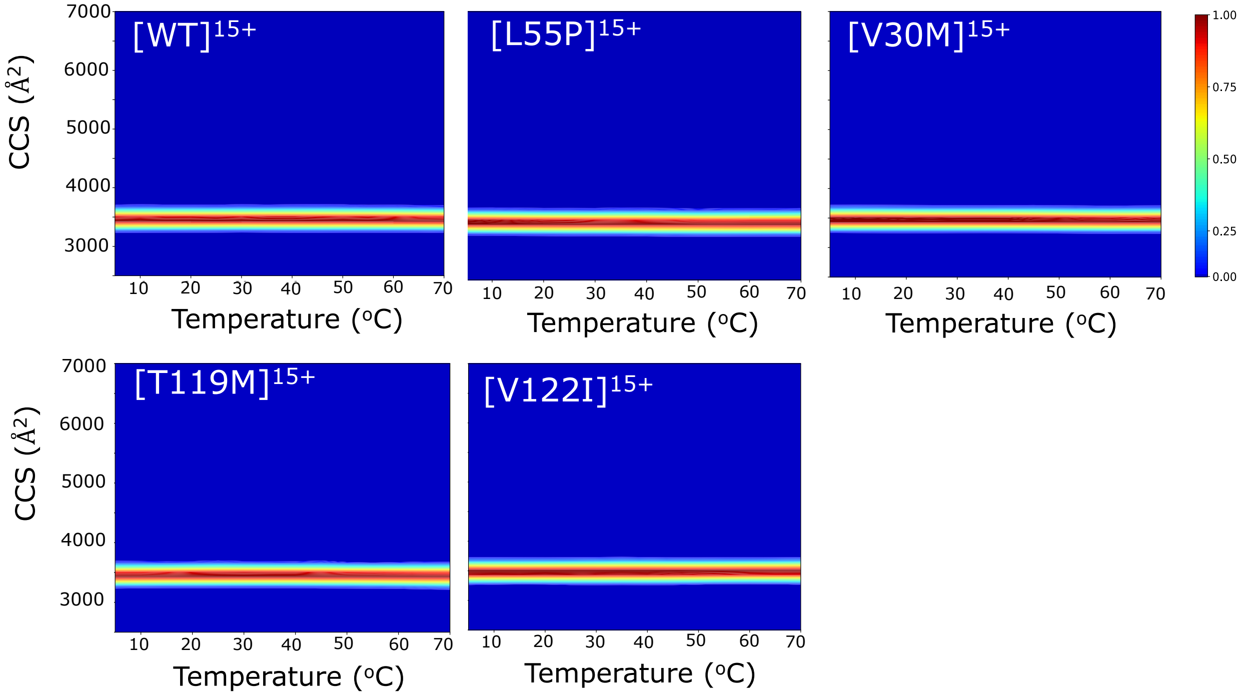

Figure S8: Variable temperature ion mobility analysis of the TTR tetramers analyzed in this study in H_2O. Notice there is no change in mobility over the range of 5-70 °C.

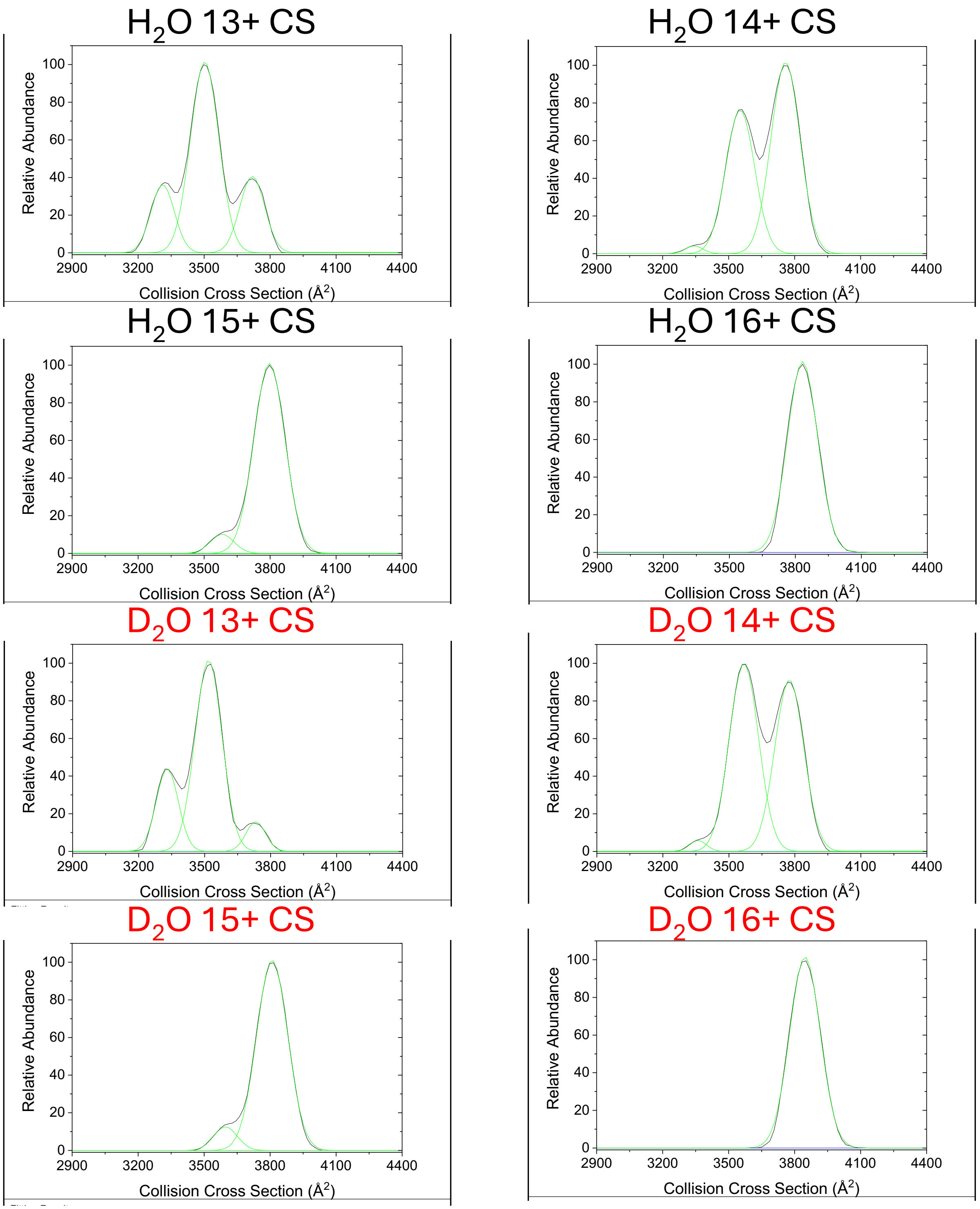

Figure S9: Fits for all TTR conformers observed in the mobilograms. Black lines correspond to the original data, and green lines correspond to the fit conformers.

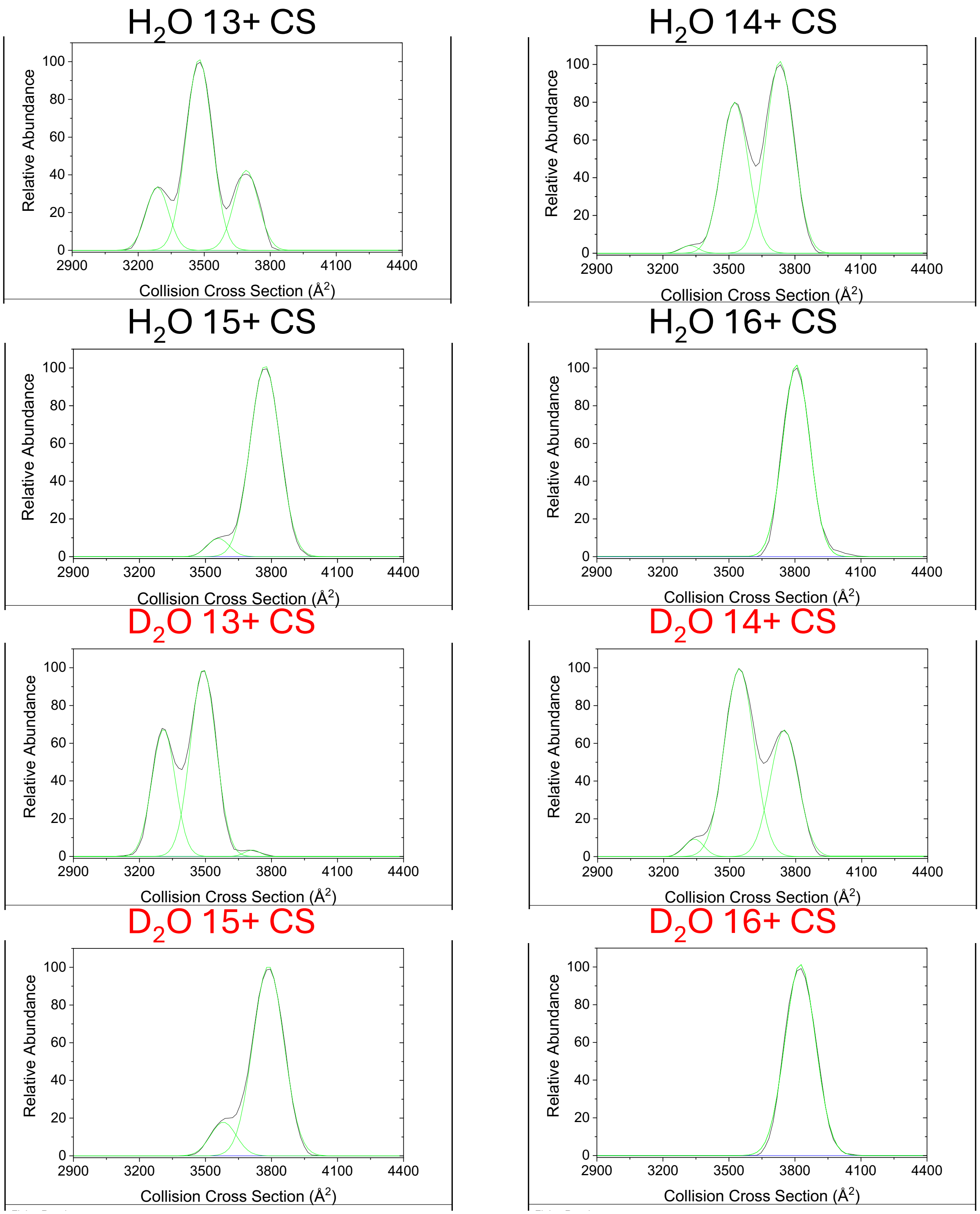

Figure S10: Fits for all TTR[L55P] conformers observed in the mobilograms. Black lines correspond to the original data, and green lines correspond to the fit conformers.

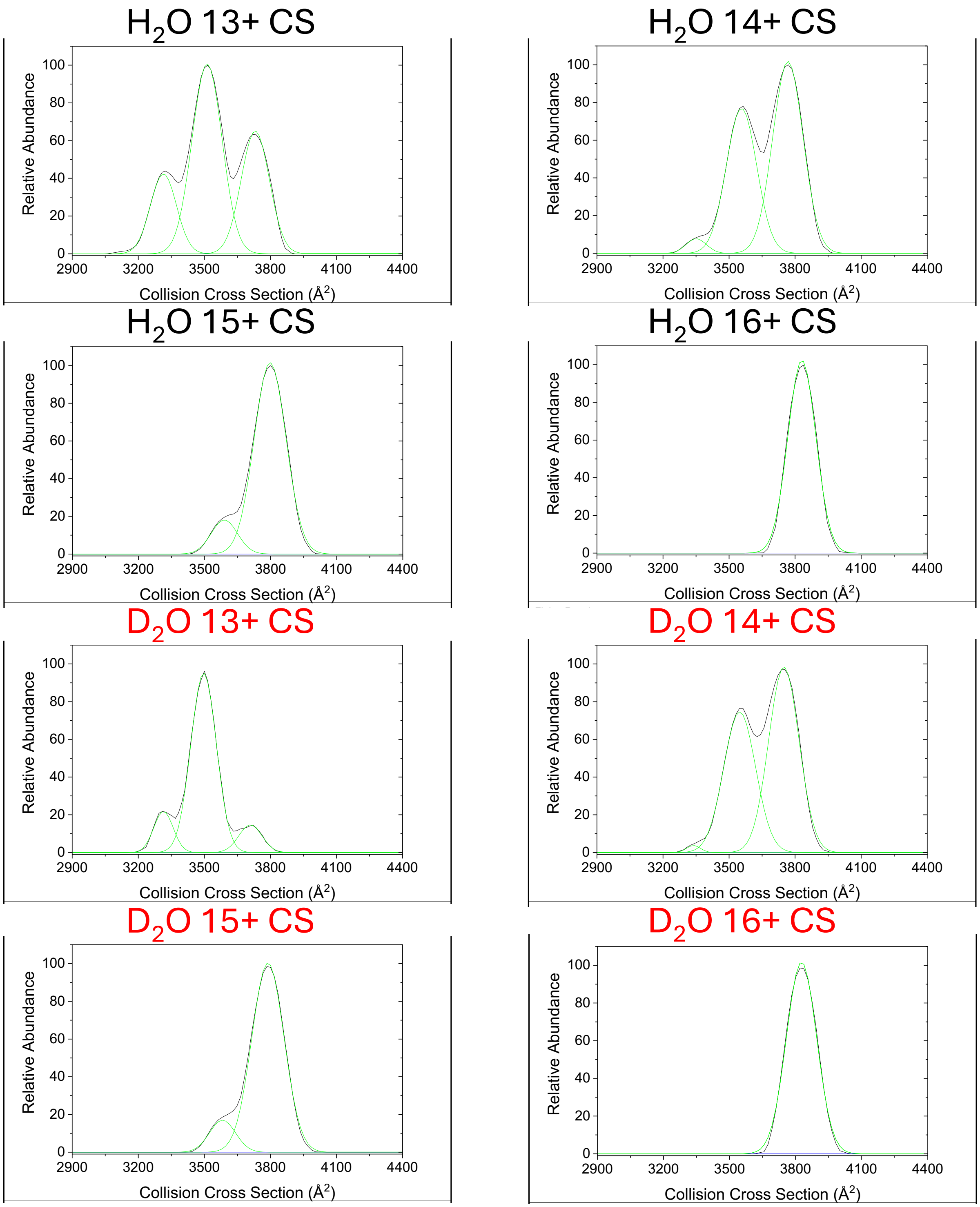

Figure S11: Fits for all TTR^{V30M} conformers observed in the mobilograms. Black lines correspond to the original data, and green lines correspond to the fit conformers.

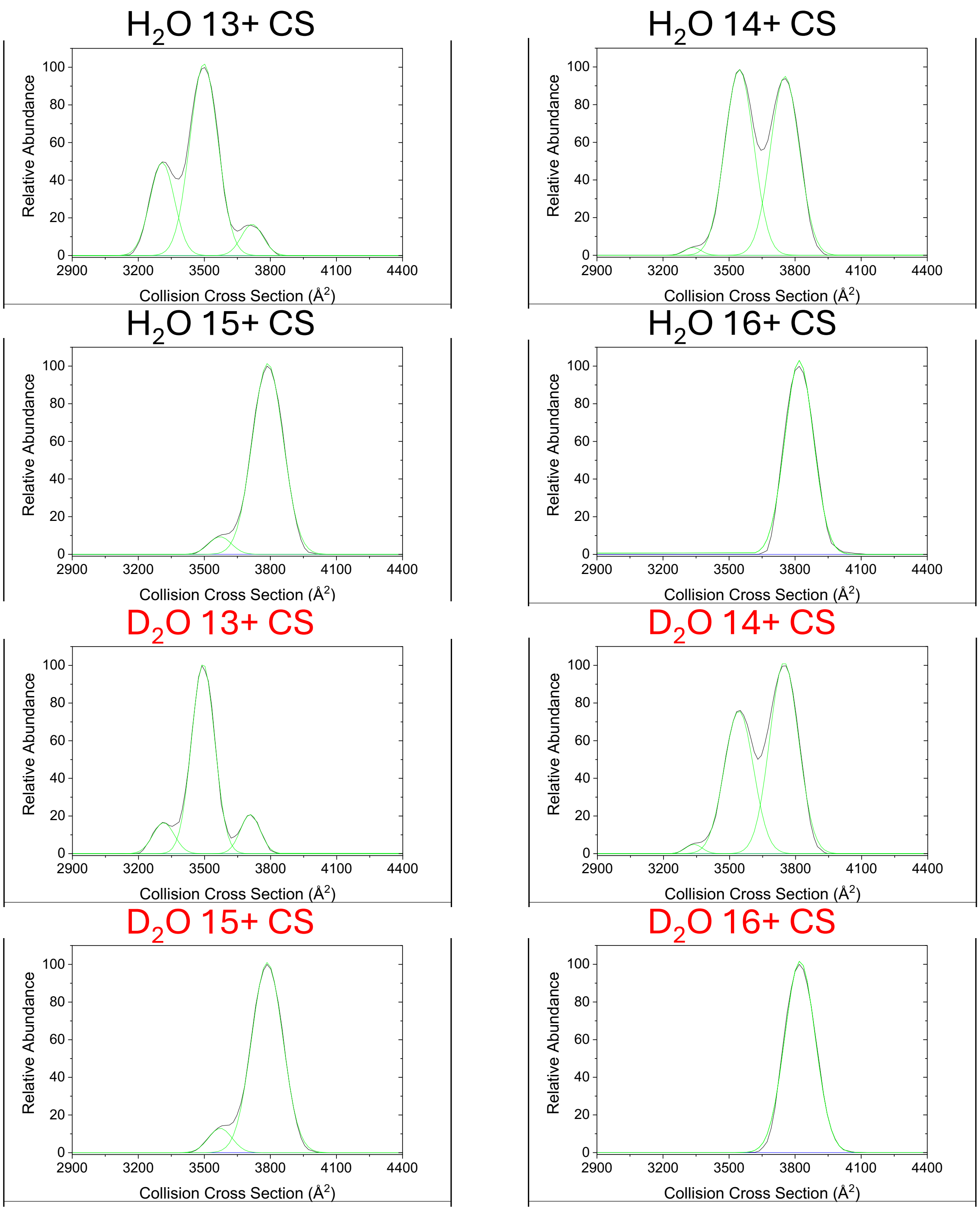

Figure S12: Fits for all TTR^{T119M} conformers observed in the mobilograms. Black lines correspond to the original data, and green lines correspond to the fit conformers.

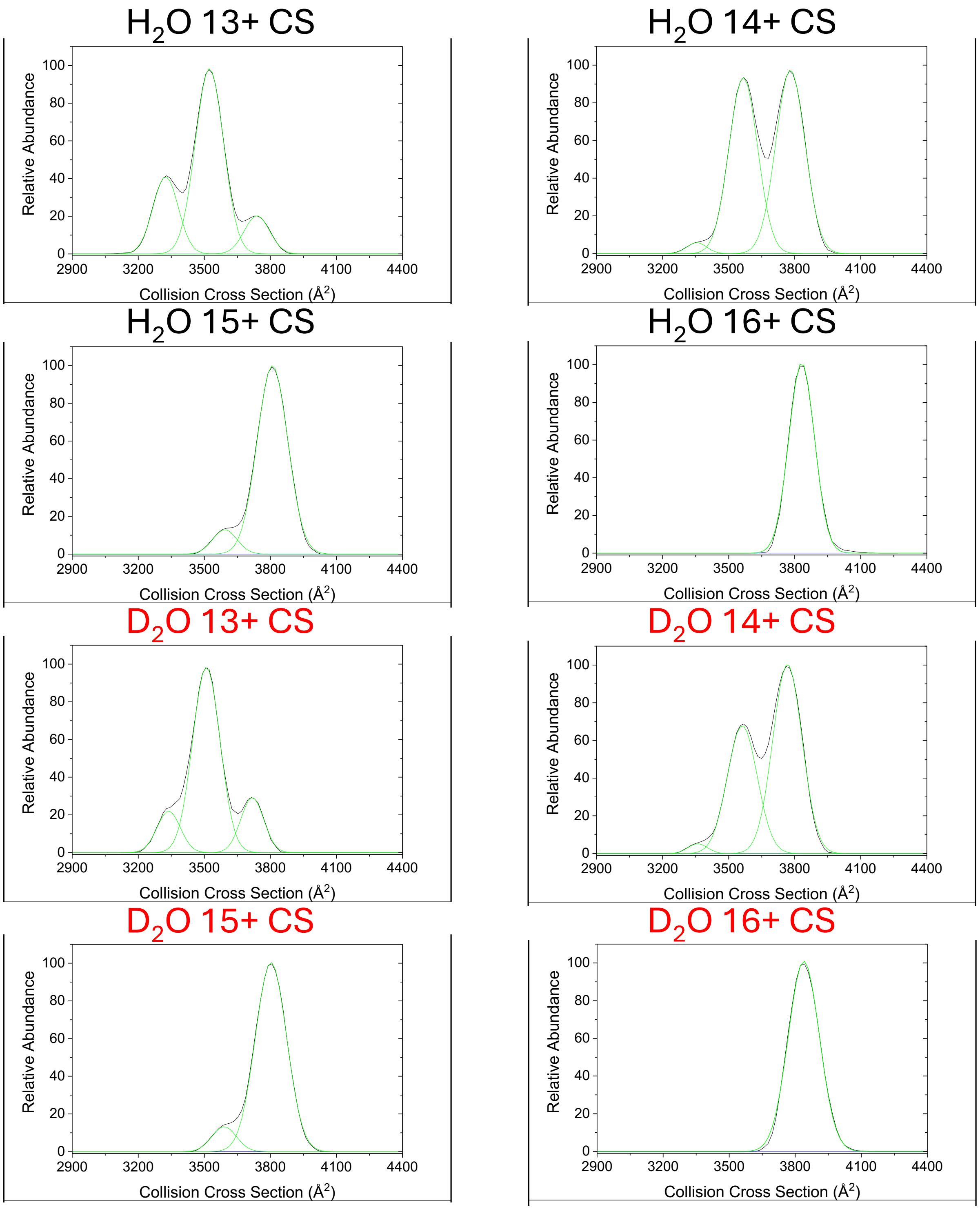

Figure S13: Fits for all TTR^{V122I} conformers observed in the mobilograms. Black lines correspond to the original data, and green lines correspond to the fit conformers.

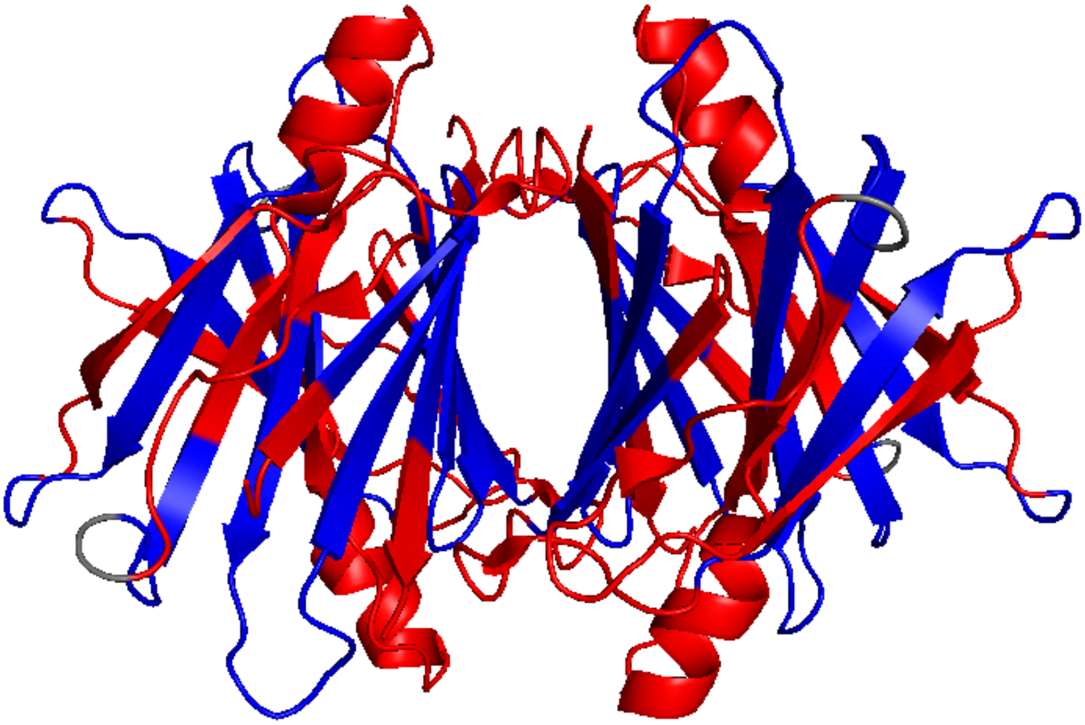

Figure S14: TTR tetrameric structure with regions that exchange hydrogen atoms with deuterium atoms readily in red and regions that do not exchange hydrogen atoms with deuterium atoms as readily in blue.

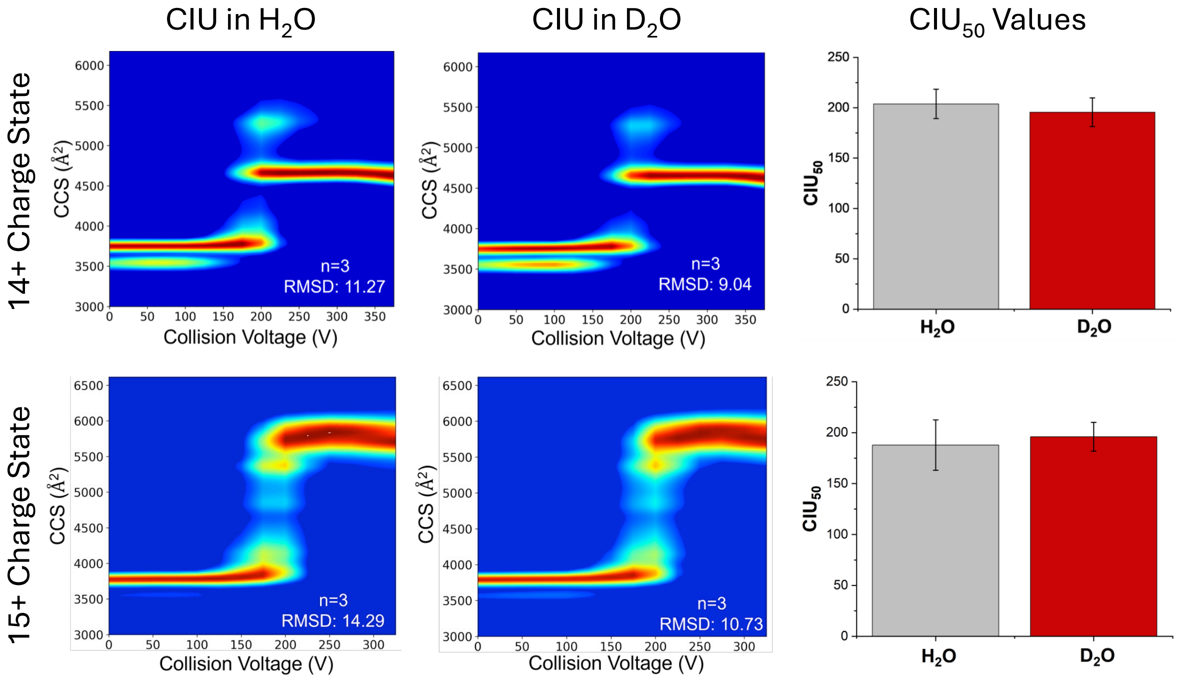

Figure S15: CIU profiles and CIU_{50} values for the 14+ and 15+ charge states TTR in H_2O and D_2O that provide evidence that isotope effects do not shift the structure of TTR significantly.

Table S1: Quantitation of conformer abundances for wtTTR					
13+ Charge State (H2O)			13+ Charge State (D2O)		
CCS (Å^2)	Area (%)	FWHM	CCS (Å^2)	Area (%)	FWHM
3311	17.42	125	3330	24.14	119
3502	58.85	152	3518	67.93	144
3719	23.73	133	3732	7.93	105
14+ Charge State (H2O)			14+ Charge State (D2O)		
CCS (Å^2)	Area (%)	FWHM	CCS (Å^2)	Area (%)	FWHM
3343	1.38	93	3360	1.69	90
3552	41.93	154	3569	50.52	157
3759	56.69	155	3775	47.78	156
15+ Charge State (H2O)			15+ Charge State (D2O)		
CCS (Å^2)	Area (%)	FWHM	CCS (Å^2)	Area (%)	FWHM
3582	7.53	127	3597	8.54	126
3797	92.47	166	3809	91.46	167
16+ Charge State (H2O)			16+ Charge State (D2O)		
CCS (Å^2)	Area (%)	FWHM	CCS (Å^2)	Area (%)	FWHM
3833	100.00	161	3847	100.00	163

Table S2: Quantitation of conformer abundances for L55P					
13+ Charge State (H2O)			13+ Charge State (D2O)		
CCS (Å^2)	Area (%)	FWHM	CCS (Å^2)	Area (%)	FWHM
3287	16.83	121	3310	37.81	125
3477	58.78	140	3490	60.92	137
3691	24.39	131	3712	1.27	82
14+ Charge State (H2O)			14+ Charge State (D2O)		
CCS (Å^2)	Area (%)	FWHM	CCS (Å^2)	Area (%)	FWHM
3323	1.38	92	3340	3.42	101
3526	41.99	145	3544	56.69	159
3731	56.63	148	3748	39.89	151
15+ Charge State (H2O)			15+ Charge State (D2O)		
CCS (Å^2)	Area (%)	FWHM	CCS (Å^2)	Area (%)	FWHM
3558	7.20	117	3581	12.91	138
3770	92.80	157	3786	87.09	165
16+ Charge State (H2O)			16+ Charge State (D2O)		
CCS (Å^2)	Area (%)	FWHM	CCS (Å^2)	Area (%)	FWHM
3806	100.00	144	3824	100.00	163

**Table S3:** Quantitation of conformer abundances for V30M					
13+ Charge State (H2O)			13+ Charge State (D2O)		
CCS ($Å^2$)	Area (%)	FWHM	CCS ($Å^2$)	Area (%)	FWHM
3314	18.09	139	3314	13.36	104
3512	49.37	160	3497	75.89	136
3731	32.54	151	3709	10.75	124
14+ Charge State (H2O)			14+ Charge State (D2O)		
CCS ($Å^2$)	Area (%)	FWHM	CCS ($Å^2$)	Area (%)	FWHM
3351	2.77	108	3339	0.93	73
3557	41.33	162	3548	43.47	170
3767	55.90	162	3749	55.59	162
15+ Charge State (H2O)			15+ Charge State (D2O)		
CCS ($Å^2$)	Area (%)	FWHM	CCS ($Å^2$)	Area (%)	FWHM
3590	12.80	141	3582	12.05	139
3798	87.20	170	3789	87.95	172
16+ Charge State (H2O)			16+ Charge State (D2O)		
CCS ($Å^2$)	Area (%)	FWHM	CCS ($Å^2$)	Area (%)	FWHM
3831	100.00	149	3827	100.00	162

**Table S4:** Quantitation of conformer abundances for T119M					
13+ Charge State (H2O)			13+ Charge State (D2O)		
CCS ($Å^2$)	Area (%)	FWHM	CCS ($Å^2$)	Area (%)	FWHM
3309	27.18	130	3314	11.02	115
3498	64.17	150	3493	72.33	123
3717	8.65	116	3707	16.65	106
14+ Charge State (H2O)			14+ Charge State (D2O)		
CCS ($Å^2$)	Area (%)	FWHM	CCS ($Å^2$)	Area (%)	FWHM
3336	1.19	86	3338	1.66	88
3547	49.85	153	3542	41.84	155
3754	48.96	153	3748	56.50	154
15+ Charge State (H2O)			15+ Charge State (D2O)		
CCS ($Å^2$)	Area (%)	FWHM	CCS ($Å^2$)	Area (%)	FWHM
3571	6.50	121	3572	8.81	126
3788	93.50	165	3786	91.19	166
16+ Charge State (H2O)			16+ Charge State (D2O)		
CCS ($Å^2$)	Area (%)	FWHM	CCS ($Å^2$)	Area (%)	FWHM
3819	100.00	152	3822	100.00	162

**Table S5:** Quantitation of conformer abundances for V122I					
13+ Charge State (H2O)			13+ Charge State (D2O)		
CCS (Å^2)	Area (%)	FWHM	CCS (Å^2)	Area (%)	FWHM
3324	23.86	135	3338	12.97	124
3523	63.66	150	3509	68.62	147
3739	12.48	135	3718	18.41	123
14+ Charge State (H2O)			14+ Charge State (D2O)		
CCS (Å^2)	Area (%)	FWHM	CCS (Å^2)	Area (%)	FWHM
3355	2.01	104	3360	2.13	103
3566	47.27	153	3562	39.85	159
3780	50.72	156	3767	58.02	156
15+ Charge State (H2O)			15+ Charge State (D2O)		
CCS (Å^2)	Area (%)	FWHM	CCS (Å^2)	Area (%)	FWHM
3593	9.48	130	3590	9.38	131
3810	90.52	163	3803	90.62	167
16+ Charge State (H2O)			16+ Charge State (D2O)		
CCS (Å^2)	Area (%)	FWHM	CCS (Å^2)	Area (%)	FWHM
3831	100.00	137	3839	100.00	167
